# Fucosylation Dynamics as a Critical Determinant of Cancer Cell Fate in Colorectal Carcinoma: Integrating Hallmark Plasticity, Microenvironmental Remodelling, and Therapeutic Resistance

**DOI:** 10.3390/biology15090689

**Published:** 2026-04-28

**Authors:** Abdulaziz Alfahed, Abdulrahman A. Alahmari, Glowi Alasiri

**Affiliations:** 1 Department of Medical Laboratory, College of Applied Medical Sciences, Prince Sattam Bin Abdulaziz University, Al Kharj 11942, Saudi Arabia; aa.alahmari@psau.edu.sa; 2 Department of Biochemistry, College of Medicine, Imam Mohammad Ibn Saud Islamic University (IMSIU), Riyadh 13317, Saudi Arabia

**Keywords:** colorectal cancer, fucosylation, cell fate determination, epithelial–mesenchymal transition (EMT), tumour microenvironment (TME), multidrug resistance, microsatellite instability (MSI), receptor tyrosine kinase (RTK) signalling, metabolic reprogramming, prognostic biomarker

## Abstract

Fucosylation, the enzymatic addition of fucose residues to glycans, modulates receptor signalling and cellular identity in the intestinal epithelium. Its role as an integrative determinant of cancer cell fate in colorectal cancer (CRC) remains undefined. Transcriptomic and clinicopathological data from 976 CRC patients across three independent cohorts (TCGA-CRC, CPTAC2-CRC, Sidra-LUMC) were analysed. A curated fucosylation gene set was used to calculate tumour fucosylation scores. Associations with histogenetic status, genomic features, microenvironmental phenotypes, drug resistance programmes, and survival were evaluated using gene set enrichment analysis, multivariable Cox regression, and integrated molecular subtyping. High-fucosylation tumours exhibited elevated epithelial differentiation, MSI-H/*BRAF*-mutant enrichment, oxidative phosphorylation dominance, the complete absence of EMT and invasion programmes, and favourable prognosis (HR = 0.633, 95% CI: 0.470–0.853, *p* = 0.003). Low-fucosylation tumours demonstrated mesenchymal phenotypes, *TP53* mutations, chromosomal instability, comprehensive multi-family RTK signalling, immune-excluded microenvironments, and poor outcomes. Distinct multidrug resistance programmes emerged: drug efflux in low-fucosylation tumours versus xenobiotic sensing, target bypass, and drug sequestration in high-fucosylation tumours. Tumour fucosylation status defines two fundamentally distinct CRC cell states with mutually exclusive engagement of invasion programmes, metabolic pathways, immune phenotypes, and resistance mechanisms. Fucosylation represents an independent prognostic biomarker and integrative determinant of cancer cell fate, with significant implications for risk stratification and personalised therapeutic strategies.

## Simple Summary

Colorectal cancer is not a single disease but a collection of different tumour types with distinct behaviours and outcomes. This study investigated how sugar modifications on cancer cell surfaces, specifically a process called fucosylation, influence tumour behaviour and patient survival. By analysing genetic data from nearly 1000 colorectal cancer patients, we discovered that tumours fall into two distinct groups based on their fucosylation levels. Patients with high fucosylation tumours survived nearly twice as long as those with low fucosylation tumours. High fucosylation tumours resembled normal, well-behaved intestinal cells, grew slowly, and retained features of healthy tissue. They were also more likely to have genetic instability at the DNA sequence level, which makes them responsive to immunotherapy. In contrast, low fucosylation tumours were aggressive, had lost their intestinal identity, and had reshaped their surrounding environment to promote invasion and suppress immune responses. These tumours relied on different mechanisms to resist chemotherapy—primarily pumping drugs out of cells—compared to high fucosylation tumours, which used more sophisticated strategies like drug sequestration and alternative survival pathways. Measuring fucosylation levels could help doctors predict which patients will have more aggressive disease and might guide treatment decisions, potentially matching patients with therapies most likely to work for their specific tumour type.

## 1. Introduction

Colorectal cancer (CRC) progression is fundamentally driven by dysregulated signalling pathways that govern cellular fate decisions between proliferation, differentiation, senescence, and death. These fate decisions are orchestrated by interconnected networks, including p53, Wnt/β-catenin, Notch, PI3K/AKT, MAPK, and TGF-β pathways, alongside epigenetic regulators and microenvironmental cues [1,2,3]. Perturbations within these signalling systems destabilize epithelial homeostasis and promote plasticity, enabling tumour cells to adopt aggressive, therapy-resistant states.

A central but underexplored regulator of these signalling networks is glycosylation. Aberrant glycosylation is a hallmark of cancer, influencing receptor activation, ligand binding, adhesion, immune recognition, and signal transduction [4]. Among glycosylation modifications, fucosylation—the enzymatic addition of fucose residues to N- and O-glycans—plays a critical role in modulating cell surface receptor signalling [5,6]. Core fucosylation, catalysed by FUT8, regulates TGF-β receptor signalling, EGFR activity, and integrin function [5,6]. O-fucosylation of the Notch receptor is indispensable for ligand binding and downstream fate determination [7,8]. These observations position fucosylation as a biochemical determinant of signalling fidelity and cellular destiny.

In the intestinal epithelium, cell fate balance between stemness and differentiation is tightly controlled by Wnt and Notch signalling gradients [2]. Disruption of this equilibrium in CRC drives dedifferentiation, epithelial–mesenchymal transition (EMT), and acquisition of stem-like phenotypes [3,9]. EMT represents a partial reprogramming event that redefines epithelial cell identity toward a mesenchymal, migratory, and therapy-resistant state. Importantly, EMT intersects with immune evasion, metabolic reprogramming, and multidrug resistance programs, underscoring its central role in tumour cell fate plasticity [10,11].

While individual fucosyltransferases have been implicated in tumour invasion and metastasis, the broader question of whether global tumour fucosylation levels stratify CRC according to cell fate states remains unresolved. Moreover, it is unclear whether variations in tumour fucosylation associate with the specific genomic contexts, immune landscapes, and therapy resistance mechanisms that collectively define cancer cell destiny.

The study aim is to investigate tumour fucosylation as an integrative determinant of cell fate states in CRC. The specific objectives are (i) to determine whether tumour fucosylation levels associate with the histogenetic status of tumour cells; (ii) to evaluate the association between fucosylation levels and tumour behaviour (adverse clinicopathological features and prognosis); (iii) to characterize the fucosylation correlates of the genomic/molecular subsets of CRC (MSI vs. MSS, molecular subtypes, molecular/genomic dichotomies); (iv) to characterize the drug resistance phenotypes of fucosylation subsets; (v) to evaluate tumour microenvironmental phenotypes, including immune-excluded, immune-desert, epithelial–mesenchymal transition (EMT), and stromal enrichment states of the fucosylation subsets of CRC; (vi) to define hallmark pathway differences between fucosylation-high and fucosylation-low CRC.

## 2. Materials and Methods

### 2.1. Study Cohorts and Data Harmonisation

This study utilised transcriptomic and clinicopathological data from three independent colorectal cancer cohorts. The Cancer Genome Atlas (TCGA-CRC) and Clinical Proteomic Tumor Analysis Consortium (CPTAC2-CRC) datasets were accessed through publicly available repositories, as previously described [1,12]. An additional cohort, the Sidra–LUMC CRC cohort [13], was also included. Only primary colorectal adenocarcinoma specimens with available transcriptomic and clinical annotations were retained for analysis. A total of 995 primary tumour samples were initially retrieved across the three datasets, comprising TCGA (*n* = 537), Sidra–LUMC (*n* = 348), and CPTAC2 (*n* = 110). Inclusion criteria comprised primary colorectal adenocarcinoma samples with available transcriptomic profiles and corresponding clinicopathological annotations. Samples lacking key clinical variables or with incomplete transcriptomic data were excluded from the analysis. Following quality control and application of inclusion and exclusion criteria, the final analytical cohort consisted of 988 tumours, distributed as follows: TCGA (*n* = 534), Sidra–LUMC (*n* = 348), and CPTAC2 (*n* = 106). Publicly available datasets were accessed via the Genomic Data Commons (TCGA) and CPTAC data portals, while the Sidra–LUMC dataset was obtained from previously published sources. Missing data were handled by case-wise exclusion for analyses requiring complete variables.

To ensure compatibility across platforms, gene identifiers were standardised, and only genes common to all three datasets were included, yielding a final set of 11,731 genes. Expression matrices were subsequently merged into a unified dataset. Batch effects attributable to cohort origin were mitigated using the ComBat algorithm implemented in the sva R package version 3.58.0 [14]. Principal component analysis was performed before and after correction to confirm successful removal of cohort-driven clustering. To quantify batch-associated variation, principal component coordinates (PC1 and PC2) were analysed to estimate the proportion of variance explained by dataset origin (R^2^).

### 2.2. Definition and Scoring of the Fucosylation Programme

A curated fucosylation gene set was constructed based on established components of GDP-fucose biosynthesis and fucosyltransferase-mediated glycosylation [15,16]. The set included GMDS, TSTA3, SLC35C1, and members of the FUT family (FUT1–FUT11, as expressed). Tumour-level fucosylation scores were calculated using two complementary approaches, standardised z-score averaging and single-sample gene set enrichment analysis, consistent with previously described transcriptomic scoring frameworks [17]. Tumours were stratified into high- and low-fucosylation subsets based on the 75th percentile of the fucosylation score, as this threshold demonstrated the strongest association with overall survival on Kaplan–Meier analysis (Supplementary Figure S1).

### 2.3. Construction of Functional and Phenotypic Modules

Functional modules were generated using reproducible Bash-executable R scripts, with module scores derived either by aggregating standardised expression values or through single-sample gene set enrichment methods [17]. To capture mechanisms of therapeutic resistance, a series of multidrug resistance modules were constructed based on established resistance pathways [18,19,20]. These included modules representing efflux pump activity, metabolic inactivation, apoptosis suppression, target bypass mechanisms, stress adaptation, xenobiotic sensing, and drug trafficking and sequestration. Additional phenotypic modules were developed to assess tumour state, including a proliferation versus epithelial–mesenchymal transition (EMT) or stromal programme axis, an epithelial differentiation index, and immune phenotype modules designed to characterise the tumour immune microenvironment. Table S1: Supplementary Materials S1 list the gene sets used to generate the functional and phenotypic modules.

### 2.4. Differential Expression and Enrichment Analyses

For pathway-level analyses, Gene Set Enrichment Analysis was conducted using curated gene sets [17]. Hallmark gene sets and curated collections were evaluated, with significance thresholds set at a false discovery rate of less than 0.25 for discovery analyses and less than 0.05 for high-confidence findings. Gene Ontology Enrichment Analysis was performed using over-representation testing frameworks [21]. Drug Ontology Enrichment Analysis was additionally conducted to identify drug classes associated with fucosylation-related transcriptional signatures, utilising curated pharmacogenomic databases.

### 2.5. Association with Tumour Progression and Phenotypic States

To determine whether fucosylation status correlates with tumour progression, associations between fucosylation scores and clinicopathological parameters—including tumour stage, nodal involvement, and metastatic status—were evaluated. Correlations between fucosylation scores and tumour state modules, epithelial differentiation index, and immune phenotype modules were also assessed to determine whether tumour fucosylation reflects an epithelial-maintaining or tumour-suppressive phenotype.

### 2.6. Statistical Analysis

All statistical analyses were performed using IBM SPSS Statistics version 29. Associations between categorical variables were examined using the chi-squared test or Fisher’s exact test, as appropriate. Continuous associations were assessed using Spearman’s or Pearson’s correlation coefficients. Comparisons of continuous variables between groups were conducted using the Mann–Whitney U test or independent-samples *t*-test. All tests were two-sided, with a *p*-value of less than 0.05 considered statistically significant. Multiple testing correction was applied using the Benjamini–Hochberg method. Missing data were handled in SPSS using case-wise (listwise) deletion for analyses requiring complete variables. Specifically, only samples with complete data for all variables included in a given analysis were retained, and cases with missing values were excluded from that analysis. No imputation of missing values was performed.

### 2.7. Data Visualisation

Visualisations were generated using SPSS outputs and the SRPlot platform, which was employed for enrichment analyses and module-based graphical representations (https://www.bioinformatics.com.cn/en, accessed on 28 February 2026) [22]. Whilst continuous fucosylation scores were compared between groups using the Mann–Whitney U test for figure visualizations, dichotomized fucosylation scores were compared to clinicopathological features using Chi-square tests.

## 3. Results

Prior to batch correction, dataset origin accounted for a substantial proportion of the variance in PCA space (R^2^ = 0.85), indicating strong cohort-driven structure. Following ComBat correction, batch-associated variance was effectively eliminated (R^2^ ≈ 0), with samples showing complete intermixing across cohorts.

### 3.1. Association of Tumour Fucosylation with Histogenetic Status and Tumour Microenvironmental Phenotypes

**Figure 1:**
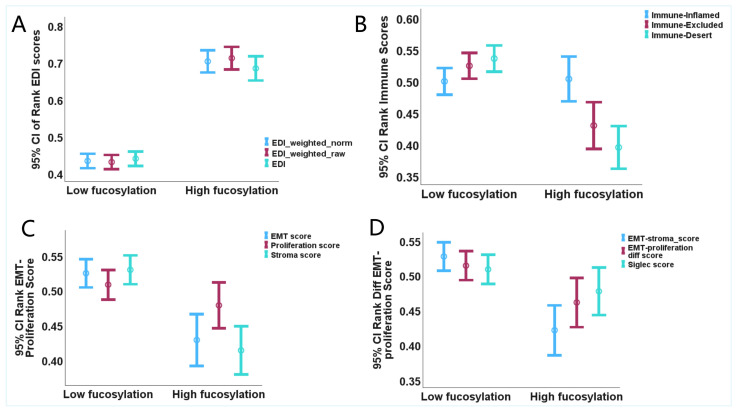
Associations between tumour fucosylation status and epithelial differentiation, immune phenotypes, and EMT–stromal programs in colorectal cancer. Error-bar plots show mean rank scores with 95% confidence intervals (CI), comparing low-fucosylation and high-fucosylation colorectal cancer subsets. (**A**) Epithelial differentiation index (EDI) scores derived using three approaches (EDI_weighted_norm, EDI_weighted_raw, and EDI) are consistently higher in high-fucosylation tumours, indicating greater epithelial differentiation compared with low-fucosylation tumours. (**B**) Immune phenotype scores show that immune-excluded and immune-desert signatures are higher in low-fucosylation tumours, whereas high-fucosylation tumours exhibit lower values for these immune states. The immune-inflamed phenotype showed no statistically significant difference between the two groups. (**C**) EMT and stromal scores are higher in low-fucosylation tumours, while high-fucosylation tumours demonstrate lower EMT and stromal activity. Proliferation scores showed a trend toward higher values in low-fucosylation tumours but did not reach statistical significance. (**D**) Composite pathway metrics (EMT–stroma score and EMT–proliferation differential score) further indicate enrichment of EMT–stromal programs in low-fucosylation tumours. The Siglec score did not differ significantly between the two fucosylation groups. Group differences between low- and high-fucosylation subsets were evaluated using the Mann–Whitney U test, with error bars representing 95% confidence intervals of the rank scores.

To investigate whether tumour fucosylation levels associate with histogenetic status and tumour microenvironmental phenotypes in colorectal cancer, we stratified tumours into high- and low-fucosylation subsets based on transcriptomic fucosylation scores and examined their relationships with epithelial differentiation, immune phenotypes, epithelial–mesenchymal transition programmes, stromal content, and Siglec signalling (Table 1, Figure 1).

#### 3.1.1. Epithelial Differentiation and Histogenetic Status

A central objective of this study was to determine whether tumour fucosylation levels associate with the histogenetic status of tumour cells—that is, the extent to which tumour cells retain epithelial differentiation and lineage commitment. To address this, we evaluated three iterations of the Epithelial Differentiation Index (EDI) between fucosylation subsets. All three EDI metrics demonstrated highly significant associations with fucosylation status (Figure 1A, Table 1). The weighted and normalised EDI score (EDI_w_norm_) was markedly higher in high-fucosylation tumours compared to their low-fucosylation counterparts (Mann–Whitney U = 140,817.5, standardized test statistic = 12.695, *p* < 0.001, FDR q < 0.001). Similarly, weighted and raw EDI score (EDI_w_raw_) (Mann–Whitney U = 142,996, standardized test statistic = 13.255, *p* < 0.001, FDR q < 0.001) and unweighted and non-normalised EDI score (EDI) (Mann–Whitney U = 136,234, standardized test statistic = 11.514, *p* < 0.001, FDR q < 0.001) were consistently and significantly elevated in high-fucosylation tumours (Figure 1A, Table 1).

These findings demonstrate that high-fucosylation tumours maintain a more differentiated, epithelial-like histogenetic status, characterised by retention of epithelial cell identity and differentiation programmes. In contrast, low-fucosylation tumours exhibit significantly reduced epithelial differentiation, indicating loss of histogenetic identity and adoption of a less differentiated, dedifferentiated cell state. The strength and consistency of these associations across multiple EDI metrics position tumour fucosylation as a robust correlate of epithelial differentiation and lineage commitment in colorectal cancer (Figure 1).

#### 3.1.2. Immune Phenotypes

Analysis of immune classification revealed striking differences between fucosylation subsets that align with their divergent histogenetic states. While Immune-Inflamed signatures showed no significant difference between groups (Mann–Whitney U = 92,221, *p* = 0.855, FDR q = 0.855), both immune-excluded and immune-desert phenotypes were significantly enriched in low-fucosylation tumours (Figure 1B, Table 1). The Immune-Excluded phenotype demonstrated significantly higher scores in low-fucosylation tumours (standardized test statistic = −4.457, *p* = 8.32 × 10^−6^, FDR q = 1.66 × 10^−5^), indicating that although immune cells may be present, they are excluded from tumour nests. Even more pronounced was the enrichment of the Immune-Desert phenotype in low-fucosylation tumours (standardized test statistic = −6.631, *p* = 3.33 × 10^−11^, FDR q = 1.67 × 10^−10^), suggesting that low-fucosylation tumours are characterised by a profound paucity of immune infiltration. These findings demonstrate that low tumour fucosylation, in conjunction with loss of epithelial differentiation, is associated with immune-suppressed microenvironmental states, whereas high-fucosylation tumours with retained epithelial identity show no enrichment for such immunosuppressive phenotypes (Table 1, Figure 1).

#### 3.1.3. Epithelial–Mesenchymal Transition and Stromal Programmes

Consistent with their reduced epithelial differentiation, examination of the tumour cell state revealed that low-fucosylation tumours exhibit a markedly mesenchymal phenotype. The EMT-score was significantly elevated in low-fucosylation compared to high-fucosylation tumours (standardized test statistic = −4.518, *p* = 6.24 × 10^−6^, FDR q = 1.56 × 10^−5^), indicating that reduced fucosylation is associated with the adoption of a mesenchymal, invasive cell state—a finding that directly aligns with the low EDI observed in this subset (Figure 1C, Table 1). The Stroma-score was also significantly higher in low-fucosylation tumours (standardized test statistic = −5.448, *p* = 5.09 × 10^−8^, FDR q = 2.55 × 10^−7^), reflecting increased stromal content, including cancer-associated fibroblasts and extracellular matrix components. The composite EMT-stroma-score, capturing both mesenchymal transformation and stromal enrichment, was likewise elevated in low-fucosylation tumours (standardized test statistic = −5.001, *p* = 5.71 × 10^−7^, FDR q = 1.9 × 10^−6^). Notably, the EMT-proliferation-diff-score, which quantifies the balance between mesenchymal and proliferative programmes, was significantly higher in low-fucosylation tumours (standardized test statistic = −2.502, *p* = 0.012, FDR q = 0.02), indicating that these tumours prioritise invasive and migratory programmes over proliferation. In contrast, the Proliferation-score showed no significant difference between fucosylation subsets (standardized test statistic = −1.403, *p* = 0.161, FDR q = 0.201), suggesting that proliferative capacity is maintained independently of fucosylation status and histogenetic state (Table 1, Figure 1).

#### 3.1.4. Siglec Signalling

Given the established role of fucosylated glycans as ligands for Siglec receptors, we evaluated whether fucosylation status associated with Siglec signalling signatures. Both raw and z-score normalised Siglec scores demonstrated no significant differences between high- and low-fucosylation tumours (Siglec-Score-Raw: standardized test statistic = −1.493, *p* = 0.135, FDR q = 0.15; Siglec-Score-Z: standardized test statistic = −1.493, *p* = 0.135, FDR q = 0.15). This finding suggests that differential fucosylation in colorectal cancer does not translate into differential engagement of Siglec-mediated immune regulatory pathways, at least at the transcriptional level, and that the immune-evasive phenotypes observed in low-fucosylation tumours are likely mediated through other mechanisms, such as EMT-associated immune exclusion.

#### 3.1.5. Summary of Phenotypic Associations

Collectively, these results reveal a coherent phenotypic dichotomy defined by tumour fucosylation status that directly addresses the study’s objectives regarding histogenesis and tumour microenvironmental phenotypes. High-fucosylation tumours maintain higher epithelial differentiation, reflecting retention of histogenetic identity, and are characterised by an epithelial phenotype with reduced stromal content and no enrichment for immune-suppressed states. In contrast, low-fucosylation tumours exhibit loss of epithelial differentiation, immune-excluded or immune-desert microenvironments, high EMT and stromal enrichment, and a cellular state favouring mesenchymal programmes over proliferation, while maintaining comparable proliferative capacity (Table 1, Figure 1).

### 3.2. Association of Tumour Fucosylation with Clinicopathological Features and Patient Prognosis

To evaluate whether tumour fucosylation levels associate with tumour behaviour and clinical outcomes in colorectal cancer, we comprehensively analysed the relationship between fucosylation status and clinicopathological parameters, survival outcomes, and prognostic independence using both categorical and continuous analytical approaches.

#### 3.2.1. Clinicopathological Features

**Figure 2:**
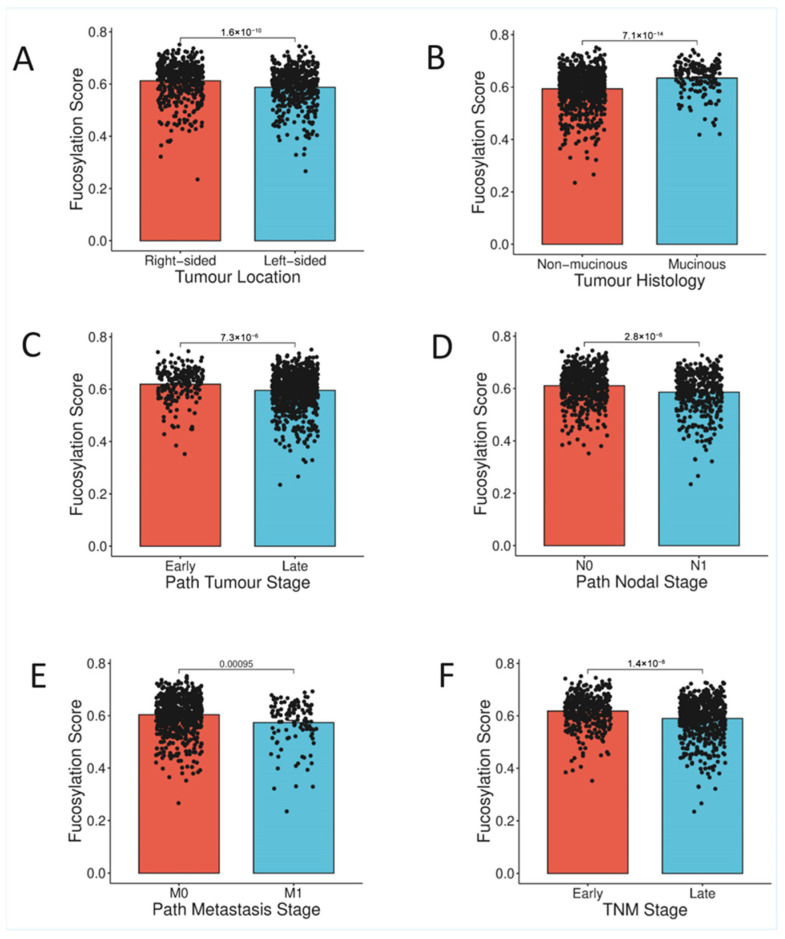
Association between tumour fucosylation score and clinicopathological characteristics in colorectal cancer. Dot-bar plots showing the distribution of tumour fucosylation scores across major clinicopathological categories in the colorectal cancer cohort. Comparisons were performed for (**A**) tumour location (right-sided vs. left-sided), (**B**) tumour histology (non-mucinous vs. mucinous), (**C**) pathological tumour stage (early vs. late), (**D**) pathological nodal stage (N0 vs. N1), (**E**) pathological metastasis stage (M0 vs. M1), and (**F**) overall TNM stage (early vs. late). Each dot represents an individual tumour sample, while bars indicate the group mean fucosylation score. Statistical differences between groups were evaluated using the Mann–Whitney U test, with corresponding *P* values shown above each comparison. Right-sided tumours exhibited modestly higher fucosylation scores than left-sided tumours. Mucinous tumours showed significantly elevated fucosylation compared with non-mucinous tumours. In contrast, reduced fucosylation scores were observed in tumours with advanced pathological stage, nodal involvement, distant metastasis, and late TNM stage, suggesting a progressive decline in tumour fucosylation with disease advancement.

Analysis of demographic and clinical characteristics revealed that fucosylation status is not confounded by age or gender. No significant differences were observed between high- and low-fucosylation groups with respect to age at diagnosis (χ^2^ = 1.516, *p* = 0.218, FDR q = 0.242) or gender distribution (χ^2^ = 2.383, *p* = 0.123, FDR q = 0.154), indicating that fucosylation-associated phenotypes are independent of these demographic variables (Table S2: Supplementary Materials S2 and Figure 2).

Striking differences emerged with respect to tumour location and histological subtype. High-fucosylation tumours were significantly more likely to arise in the right colon, whereas low-fucosylation tumours predominated in the left colon and rectum (χ^2^ = 36.946, *p* = 1.21 × 10^−9^, FDR q = 4.54 × 10^−9^). This anatomical dichotomy suggests potential links between fucosylation status and the distinct developmental origins and microenvironmental contexts of right-sided versus left-sided colorectal cancers. Furthermore, mucinous adenocarcinoma histology was markedly enriched in the high-fucosylation subset (χ^2^ = 68.106, *p* = 1.55 × 10^−16^, FDR q = 1.74 × 10^−15^), indicating that high fucosylation may associate with this distinct histological pathway of colorectal carcinogenesis (Table S2: Supplementary Materials S2, Figure 2A,B).

#### 3.2.2. Association with Tumour Progression and Aggressive Behaviour

Examination of pathological staging parameters revealed that low-fucosylation tumours exhibit a more aggressive phenotype, with adverse clinicopathological features (Table S2: Supplementary Materials S2, Figure 2C–F). Pathological T stage was significantly associated with fucosylation status (χ^2^ = 5.134, *p* = 0.023, FDR q = 0.031), with low-fucosylation tumours demonstrating deeper local invasion. Nodal involvement was also significantly more frequent in low-fucosylation tumours (χ^2^ = 4.376, *p* = 0.036, FDR q = 0.045), indicating greater propensity for lymphatic dissemination. Most notably, distant metastasis at presentation was significantly enriched in the low-fucosylation subset (χ^2^ = 8.751, *p* = 0.003, FDR q = 0.005), with 97 of 594 evaluable low-fucosylation patients presenting with metastatic disease compared to only 16 of 202 high-fucosylation patients. Consistent with these individual stage components, overall stage demonstrated a significant association with fucosylation status (χ^2^ = 6.29, *p* = 0.012, FDR q = 0.017), with early-stage (I–II) tumours enriched in the high-fucosylation subset and advanced-stage (III–IV) tumours predominating in the low-fucosylation group.

These findings establish a clear association between low tumour fucosylation and the adverse clinicopathological features indicative of aggressive tumour behaviour—deep local invasion, nodal metastasis, distant dissemination, and advanced overall stage—whereas high fucosylation defines a subset of tumours with indolent behaviour and favourable pathological characteristics (Table S2: Supplementary Materials S2, Figure 2C–F).

#### 3.2.3. Survival Analysis: Kaplan–Meier Estimation

**Figure 3:**
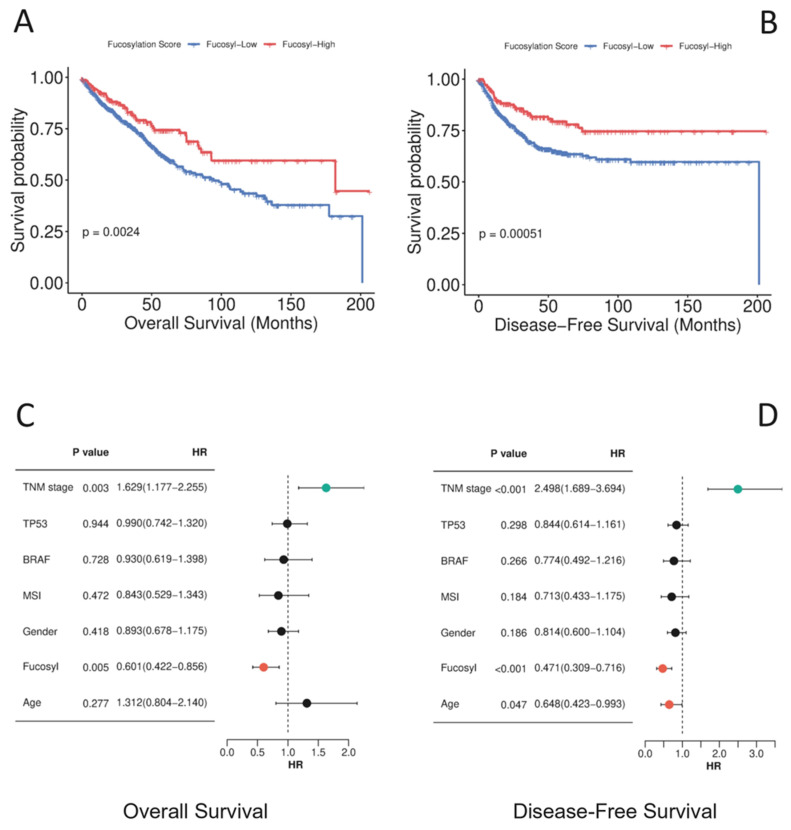
Prognostic impact of tumour fucosylation in colorectal cancer. Kaplan–Meier survival curves and multivariable Cox regression analyses evaluating the prognostic significance of tumour fucosylation score in the colorectal cancer cohort. **Top panels**: Kaplan–Meier survival curves comparing patients with fucosylation-high and fucosylation-low tumours for (**A**) overall survival (OS) and (**B**) disease-free survival (DFS). Patients with high tumour fucosylation demonstrated significantly improved survival outcomes compared with those with low fucosylation. Log-rank test P values are indicated on the plots. **Bottom panels**: Forest plots showing results from multivariable Cox proportional hazards models for (**C**) OS and (**D**) DFS. Covariates included TNM stage, *TP53* mutation status, *BRAF* mutation status, microsatellite instability (MSI) status, gender, age, and tumour fucosylation score. Hazard ratios (HRs) and 95% confidence intervals are displayed for each variable. High tumour fucosylation remained an independent protective prognostic factor for both OS and DFS after adjustment for clinical and molecular covariates. TNM stage was the strongest adverse prognostic factor, while other molecular variables did not reach statistical significance in the multivariable models.

To translate these pathological associations into clinical outcomes, we performed Kaplan–Meier survival analysis comparing overall survival and disease-free survival between fucosylation subsets (Table S3: Supplementary Materials S3, Figure 3A,B). Patients with high-fucosylation tumours demonstrated significantly better overall survival compared to those with low-fucosylation tumours (log-rank *p* = 0.002). The median overall survival for the low-fucosylation group was 94.5 months (95% C.I. = 70.8 − 118.24), whereas the high-fucosylation group exhibited a median survival of 181.9 months (95% C.I. = 23.6 − 340.1) —representing nearly double the survival time. The survival advantage was even more pronounced for disease-free survival (log-rank *p* = 0.0005), with the high-fucosylation group not reaching median survival at last follow-up, compared to a median disease-free survival of 201.1 months in the low-fucosylation group. Censoring rates were higher in the high-fucosylation group for both overall survival (78% vs. 70%) and disease-free survival (83% vs. 73%), indicating that these patients were more likely to be alive and recurrence-free at study conclusion.

#### 3.2.4. Univariate and Multivariable Cox Regression Analysis

To quantify the prognostic impact of fucosylation status, we performed univariate Cox regression analysis. High fucosylation was associated with a significantly reduced hazard of death (HR = 0.633, 95% CI: 0.470 − 0.853, *p* = 0.003), corresponding to a 36.7% reduction in mortality risk compared to low-fucosylation tumours. For comparison, advanced overall stage (III–IV vs. I–II) conferred a more than doubling of mortality risk (HR = 2.045, 95% CI: 1.514 − 2.762, *p* = 3.11 × 10^−6^), consistent with its established role as a powerful prognostic factor.

Crucially, in multivariable Cox regression analysis, adjusting for age, gender, *TP53* mutation, *BRAF* mutation, MSI status, and overall stage, high fucosylation remained an independent predictor of improved survival (Figure 3C,D). Patients with high-fucosylation tumours had a significantly reduced hazard of death compared to those with low-fucosylation tumours (HR = 0.601, 95% CI: 0.422 − 0.856, *p* = 0.005), corresponding to a 39.9% reduction in mortality risk. Overall stage was also independently associated with survival (HR = 1.629, 95% CI: 1.177 − 2.255, *p* = 0.003), while age, gender, *TP53* mutation, *BRAF* mutation, and MSI status were not significant predictors in this model. These findings establish that the survival benefit associated with high fucosylation is not merely a reflection of earlier stage at presentation but represents an independent biological effect, with high fucosylation conferring approximately a 39.9% reduction in mortality risk after accounting for established prognostic factors (Table S3: Supplementary Materials S3, Figure 3).

#### 3.2.5. Dose–Response Relationship: Continuous Fucosylation Score Analysis

To further validate the prognostic significance of fucosylation and explore potential dose–response relationships, we analysed fucosylation as a continuous variable using single-sample Gene Set Enrichment Analysis scores. This approach revealed an even stronger association with survival outcomes. For overall survival, each one-unit increase in the continuous fucosylation score was associated with a 90.8% reduction in mortality risk (HR = 0.092, 95% CI: 0.021 − 0.408, *p* = 0.002). For disease-free survival, each unit increase conferred a 92.6% reduction in recurrence risk (HR = 0.074, 95% CI: 0.014 − 0.395, *p* = 0.002). This dose–response relationship—wherein progressively higher fucosylation scores are associated with incrementally better outcomes—provides powerful evidence for the biological relevance of fucosylation as a continuous determinant of tumour behaviour, rather than merely a dichotomous classifier (Table S3: Supplementary Materials S3).

#### 3.2.6. Integration with Histogenetic and Microenvironmental Phenotypes

The survival benefits observed in high-fucosylation tumours align perfectly with their favourable clinicopathological profile—early stage, node-negative status, absence of metastasis—and with our previous findings demonstrating that high-fucosylation tumours retain epithelial differentiation (high EDI), exhibit low EMT and stromal signatures, and show no enrichment for immune-suppressed phenotypes. Conversely, the poor outcomes in low-fucosylation tumours are consistent with their adverse pathological features, loss of epithelial differentiation, mesenchymal transformation, stromal enrichment, and immune-excluded or immune-desert microenvironments (Figure 1, Table 1, Figure 2, Table S2: Supplementary Materials S2, Table S3: Supplementary Materials S3). This integrated analysis positions fucosylation status as a central determinant of the indolent versus aggressive dichotomy in colorectal cancer, capturing a fundamental biological heterogeneity that translates into meaningful differences in patient outcomes.

### 3.3. Association of Tumour Fucosylation with Genomic and Molecular Subsets of Colorectal Cancer

**Figure 4:**
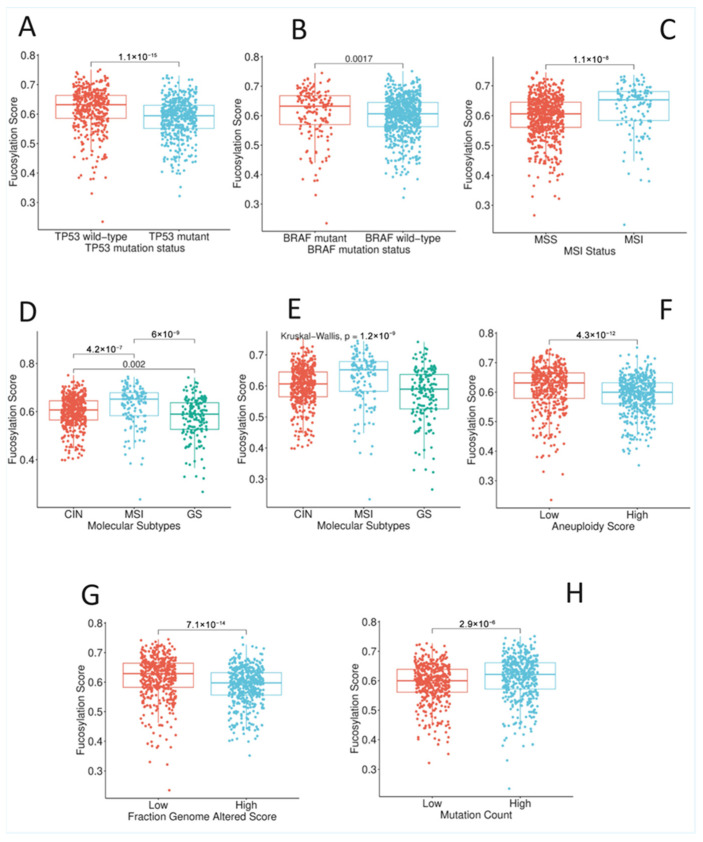
Association of tumour fucosylation with genomic alterations and molecular subtypes in colorectal cancer. Box-and-jitter plots showing the distribution of tumour fucosylation scores across key genomic and molecular features of colorectal cancer. Comparisons were performed according to (**A**) *TP53* mutation status (wild-type vs. mutant), (**B**) *BRAF* mutation status (mutant vs. wild-type), (**C**) microsatellite instability (MSI) status (microsatellite stable [MSS] vs. MSI), (**D**,**E**) molecular subtypes defined by chromosomal instability (CIN), microsatellite instability (MSI), and genome stable (GS) groups, (**F**) aneuploidy score (low vs. high), (**G**) fraction genome altered (FGA) (low vs. high), and (**H**) tumour mutation count (low vs. high). Each dot represents an individual tumour sample, while box plots indicate the median and interquartile range. Tumours harbouring *TP53* mutations showed modestly lower fucosylation scores compared with *TP53* wild-type tumours. *BRAF*-mutant tumours also demonstrated increased fucosylation relative to *BRAF* wild-type cases. MSI tumours exhibited significantly higher fucosylation scores compared with MSS tumours. Among molecular subtypes, MSI tumours displayed the highest fucosylation levels, whereas GS tumours exhibited the lowest, with CIN tumours showing intermediate values; group differences were assessed using the Kruskal–Wallis test followed by pairwise comparisons. In addition, tumours with high aneuploidy scores and high fraction genome altered (FGA) demonstrated significantly lower fucosylation scores, whereas tumours with higher mutation burden showed increased fucosylation. Statistical significance values for each comparison are indicated above the plots.

To characterise the molecular landscape underlying fucosylation-associated phenotypic divergence, we comprehensively analysed the relationship between tumour fucosylation status and key genomic alterations, microsatellite instability, molecular subtypes, and genomic instability metrics in colorectal cancer (Table S4: Supplementary Materials S4, Figure 4).

#### 3.3.1. Driver Gene Mutations

Analysis of canonical colorectal cancer driver genes revealed striking and opposing associations with fucosylation status (Table S4: Supplementary Materials S4, Figure 4A). *TP53* mutation status demonstrated a highly significant dichotomy between fucosylation subsets (χ^2^ = 62.001, *p* = 3.43 × 10^−15^, FDR q = 2.57 × 10^−14^). *TP53* mutations were markedly enriched in low-fucosylation tumours, with 400 of 658 evaluable low-fucosylation cases harbouring *TP53* mutations compared to only 66 of 219 high-fucosylation cases. This strong association positions low-fucosylation tumours within the chromosomal instability pathway of colorectal carcinogenesis, which is typically characterised by *TP53* mutations, aneuploidy, and adverse outcomes.

Conversely, *BRAF* mutations demonstrated a striking enrichment in high-fucosylation tumours (χ^2^ = 26.373, *p* = 2.81 × 10^−7^, FDR q = 1.05 × 10^−6^). Among the evaluable cases, 66 of 219 high-fucosylation tumours harboured *BRAF* mutations, compared to only 96 of 658 low-fucosylation tumours (Table S4: Supplementary Materials S4, Figure 4B). This association aligns with the predominance of high-fucosylation tumours in the right colon, as *BRAF*-mutant colorectal cancers are known to arise more frequently in the proximal colon and are associated with distinct clinicopathological features, including microsatellite instability and mucinous histology—both of which we observed to be enriched in the high-fucosylation subset (Table S4: Supplementary Materials S4, Figure 4).

#### 3.3.2. Microsatellite Instability Status

Consistent with the *BRAF* mutation findings, microsatellite instability status demonstrated a highly significant association with fucosylation (χ^2^ = 57.363, *p* = 3.62 × 10^−14^, FDR q = 2.03 × 10^−13^) (Table S4: Supplementary Materials S4, Figure 4C). Microsatellite instability-high (MSI-H) tumours were markedly enriched in the high-fucosylation subset, with 68 of 218 evaluable high-fucosylation cases exhibiting MSI compared to only 60 of 618 low-fucosylation cases. Conversely, microsatellite stable (MSS) tumours predominated in the low-fucosylation subset, comprising 558 of 618 cases (Table S4: Supplementary Materials S4, Figure 4C).

#### 3.3.3. Molecular Subtypes

To further resolve the molecular identity of fucosylation-defined subsets, we examined their distribution across established colorectal cancer molecular subtypes (Table S4: Supplementary Materials S4, Figure 4D,E). The association between fucosylation status and molecular subtype was highly significant (χ^2^ = 55.681, *p* = 8.11 × 10^−13^, FDR q = 7.6 × 10^−12^), revealing distinct subtype enrichment patterns. Low-fucosylation tumours were predominantly classified as Epithelial/CIN (368 of 569 evaluable cases) or Mesenchymal/EMT/GS (134 of 569 cases), consistent with their *TP53* mutation-enriched, MSS-predominant profile and our previous observations of high EMT and stromal scores in this subset. The Epithelial/CIN subtype, characterised by chromosomal instability and epithelial morphology, aligns with the proliferative capacity retained in low-fucosylation tumours, while the Mesenchymal/EMT/GS subtype directly corresponds to their high EMT scores, stromal enrichment, and aggressive behaviour.

In striking contrast, high-fucosylation tumours were overwhelmingly enriched in the Hypermutated/MSI subtype (70 of 201 evaluable cases), with smaller proportions classified as Epithelial/CIN (104 cases) and only 27 cases falling into the Mesenchymal/EMT/GS subtype (Table S4: Supplementary Materials S4, Figure 4D,E). The predominance of the Hypermutated/MSI subtype in high-fucosylation tumours integrates perfectly with their *BRAF* mutation enrichment, MSI-H status, right-sided predominance, mucinous histology, and favourable prognosis. This subtype is characterised by high mutation burden, immune infiltration, and better outcomes—features that align with our observations of retained epithelial differentiation, the absence of immune suppression, and prolonged survival in high-fucosylation patients (Table S4: Supplementary Materials S4, Figure 4).

#### 3.3.4. Genomic Instability Metrics

The analysis of genomic instability metrics revealed profound differences between fucosylation subsets that further elucidate their divergent molecular pathogenesis. Aneuploidy scores demonstrated a highly significant association (χ^2^ = 74.542, *p* = 5.94 × 10^−18^, FDR q = 1.34 × 10^−16^), with high aneuploidy markedly enriched in low-fucosylation tumours (410 of 473 evaluable cases with high aneuploidy) compared to high-fucosylation tumours (only 63 of 230 cases) (Table S4: Supplementary Materials S4, Figure 4E,F). Similarly, the fraction of genome altered (FGA), a continuous measure of chromosomal copy number alterations, showed a highly significant association (χ^2^ = 70.807, *p* = 3.94 × 10^−17^, FDR q = 6.65 × 10^−16^), with high FGA observed in 394 of 454 evaluable low-fucosylation tumours compared to only 60 of 230 high-fucosylation tumours. These findings demonstrate that low-fucosylation tumours are characterised by profound chromosomal instability, with high aneuploidy and extensive genomic alterations—features typical of the conventional chromosomal instability pathway of colorectal carcinogenesis and consistent with their *TP53* mutation-enriched, MSS-predominant profile. In contrast, high-fucosylation tumours exhibit genomic stability with low aneuploidy and limited copy number alterations, aligning with their hypermutated MSI-H profile, wherein genomic instability manifests at the nucleotide level rather than the chromosomal level (Table S4: Supplementary Materials S4, Figure 4E,F).

#### 3.3.5. Mutation Burden

Consistent with the MSI and aneuploidy findings, mutation count analysis revealed a significant association with fucosylation status (χ^2^ = 30.896, *p* = 2.72 × 10^−8^, FDR q = 1.36 × 10^−7^). High mutation count, characteristic of the hypermutated phenotype, was enriched in high-fucosylation tumours, whereas low mutation count predominated in low-fucosylation tumours (Table S4: Supplementary Materials S4, Figure 4H). This pattern reflects the fundamental dichotomy between the hypermutated MSI pathway (high fucosylation) and the chromosomally unstable MSS pathway (low fucosylation), with the former characterised by high mutation burden but chromosomal stability, and the latter by low mutation burden but extensive chromosomal alterations (Table S4: Supplementary Materials S4, Figure 4H).

#### 3.3.6. Integrated Molecular Portrait

Collectively, these analyses reveal the two fundamentally distinct molecular archetypes defined by fucosylation status. High-fucosylation tumours correspond to the hypermutated MSI pathway of colorectal carcinogenesis, characterised by *BRAF* mutations, microsatellite instability, low aneuploidy, low fraction of genome altered, high mutation count, and classification within the Hypermutated/MSI molecular subtype. This molecular profile aligns with their right-sided predominance, mucinous histology, retained epithelial differentiation, favourable clinicopathological features, and excellent prognosis—collectively defining an indolent, less aggressive disease biology.

In striking contrast, low-fucosylation tumours correspond to the conventional chromosomal instability pathway, characterised by *TP53* mutations, microsatellite stability, high aneuploidy, extensive copy number alterations, low mutation count, and classification within either the Epithelial/CIN or Mesenchymal/EMT/GS molecular subtypes. This molecular architecture aligns with their left-sided predominance, loss of epithelial differentiation, mesenchymal transformation, stromal enrichment, adverse clinicopathological features, and poor prognosis—collectively defining an aggressive, invasive disease biology (Table S4: Supplementary Materials S4, Figure 4).

### 3.4. Association of Tumour Fucosylation with Multidrug Resistance Phenotypes

**Figure 5:**
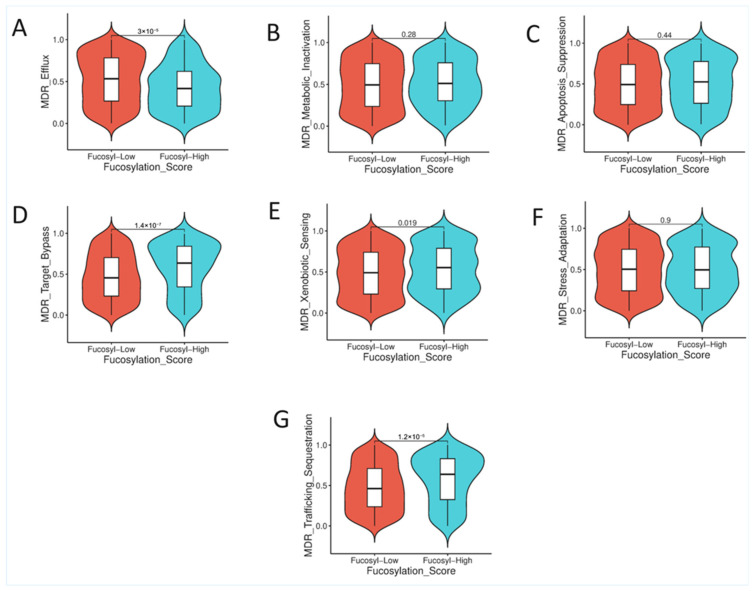
Association between tumour fucosylation status and multidrug resistance (MDR) programs in colorectal cancer. Violin plots with embedded boxplots compare multidrug resistance (MDR) functional scores between low-fucosylation and high-fucosylation colorectal cancer tumours. The distributions illustrate the density of samples, while boxplots indicate the median and interquartile range. *p*-values shown above each panel were calculated using the Mann–Whitney U test. (**A**) Drug efflux scores are higher in low-fucosylation tumours. In contrast, high-fucosylation tumours exhibit significantly higher scores for several MDR mechanisms, including (**D**) target bypass, (**E**) xenobiotic sensing, and (**G**) drug trafficking/sequestration, suggesting enhanced activation of adaptive drug resistance pathways. No statistically significant differences were observed for (**B**) metabolic inactivation, (**C**) apoptosis suppression, or (**F**) stress adaptation scores between the two fucosylation groups. Overall, these findings indicate that tumour fucosylation status is associated with distinct MDR strategy profiles, with high-fucosylation tumours showing enrichment of pathway-level mechanisms that facilitate therapeutic evasion.

To characterise the drug resistance phenotypes of fucosylation-defined subsets in colorectal cancer, we comprehensively evaluated the association between tumour fucosylation status and seven distinct multidrug resistance mechanisms, encompassing efflux pump activity, metabolic inactivation, apoptosis suppression, target bypass signalling, stress adaptation, xenobiotic sensing, and drug trafficking or sequestration. This analysis revealed a striking dichotomy in resistance mechanism engagement between high- and low-fucosylation tumours, with each subset exhibiting preferential activation of distinct, non-overlapping resistance programmes (Table S5: Supplementary Materials S5, Figure 5).

#### 3.4.1. Drug Efflux: A Resistance Mechanism Enriched in Low-Fucosylation Tumours

The analysis of drug efflux capacity, mediated by ATP-binding cassette transporters such as P-glycoprotein and other multidrug resistance proteins, demonstrated a highly significant association with fucosylation status (Mann–Whitney U = 75,311, standardized test statistic = −4.172, *p* = 3.02 × 10^−5^, FDR q = 7.05 × 10^−5^). Drug efflux signatures were significantly elevated in low-fucosylation tumours compared to their high-fucosylation counterparts (Table S5: Supplementary Materials S5, Figure 5A). This finding indicates that low-fucosylation tumours possess an enhanced capacity for active extrusion of chemotherapeutic agents, representing a classical mechanism of multidrug resistance that would limit intracellular drug accumulation and reduce therapeutic efficacy. The enrichment of this mechanism in low-fucosylation tumours aligns with their adverse clinicopathological features, aggressive behaviour, and poor prognosis, suggesting that these tumours may be intrinsically resistant to conventional chemotherapy through active drug elimination (Table S5: Supplementary Materials S5, Figure 5A).

#### 3.4.2. Target Bypass Signalling: A Resistance Mechanism Enriched in High-Fucosylation Tumours

In striking contrast to the drug efflux findings, target bypass signalling demonstrated a highly significant enrichment in high-fucosylation tumours (Mann–Whitney U = 111,946, standardized test statistic = 5.261, *p* = 1.43 × 10^−7^, FDR q = 5.01 × 10^−7^). This mechanism involves the activation of alternative survival pathways that circumvent the need for the original drug target, allowing tumour cells to maintain proliferation and survival despite targeted therapeutic intervention. The enrichment of this resistance programme in high-fucosylation tumours indicates that these cancers, despite their favourable clinicopathological features and excellent prognosis, may possess an intrinsic capacity to engage compensatory signalling networks when challenged with pathway-directed therapies. This finding has important therapeutic implications, suggesting that high-fucosylation tumours, while generally indolent, may require combination strategies to prevent adaptive resistance through target bypass (Table S5: Supplementary Materials S5, Figure 5D).

#### 3.4.3. Xenobiotic Sensing: Enhanced Detection Capacity in High-Fucosylation Tumours

Xenobiotic sensing, encompassing the molecular machinery that detects and responds to foreign compounds including chemotherapeutic agents, was significantly elevated in high-fucosylation tumours (Mann–Whitney U = 100,604, standardized test statistic = 2.341, *p* = 0.019, FDR q = 0.027) (Table S5: Supplementary Materials S5, Figure 5E). This finding indicates that high-fucosylation tumours possess enhanced capacity for detecting and initiating responses to drug exposure. While xenobiotic sensing can contribute to resistance by triggering detoxification programmes, it may also render tumours more responsive to therapy by enabling drug recognition and subsequent engagement of cell death pathways. The co-enrichment of xenobiotic sensing with target bypass signalling in high-fucosylation tumours suggests a coordinated resistance programme focused on drug recognition and adaptive survival pathway activation, rather than the physical drug elimination strategy employed by low-fucosylation tumours (Table S5: Supplementary Materials S5, Figure 5E).

#### 3.4.4. Drug Trafficking and Sequestration: Enhanced Compartmentalisation in High-Fucosylation Tumours

Analysis of drug trafficking and sequestration mechanisms, which involve the compartmentalisation of chemotherapeutic agents away from their intracellular targets into lysosomes, endosomes, or other organelles, revealed significant enrichment in high-fucosylation tumours (Mann–Whitney U = 108,486, standardized test statistic = 4.37, *p* = 1.24 × 10^−5^, FDR q = 2.17 × 10^−5^) (Table S5: Supplementary Materials S5, Figure 5G). This finding indicates that high-fucosylation tumours possess enhanced capacity for physical sequestration of drugs into subcellular compartments where they cannot access their intended targets. Together with xenobiotic sensing and target bypass signalling, this mechanism completes a triad of resistance programmes preferentially engaged by high-fucosylation tumours—focused on drug recognition, adaptive survival signalling, and subcellular drug compartmentalisation (Table S5: Supplementary Materials S5, Figure 5G).

#### 3.4.5. Resistance Mechanisms with No Differential Engagement

Several multidrug resistance mechanisms showed no significant association with fucosylation status. Metabolic inactivation, involving enzymatic modification and detoxification of chemotherapeutic agents, did not differ between groups (Mann–Whitney U = 95,689, standardized test statistic = 1.075, *p* = 0.282, FDR q = 0.329) (Table S5: Supplementary Materials S5, Figure 5B). Apoptosis suppression, encompassing anti-apoptotic BCL2 family members and inhibitors of apoptosis proteins, showed no differential engagement (Mann–Whitney U = 94,518, standardized test statistic = 0.774, *p* = 0.439, FDR q = 0.439). Stress adaptation programmes, including oxidative stress response and unfolded protein response pathways, were similarly expressed across fucosylation subsets (Mann–Whitney U = 91,981.5, standardized test statistic = 0.12, *p* = 0.904, FDR q = 0.904). (Table S5: Supplementary Materials S5, Figure 5C). The absence of differential engagement in these mechanisms suggests that fucosylation status does not globally influence all resistance programmes but rather specifies-distinct, non-overlapping resistance strategies in each subset (Table S5: Supplementary Materials S5, Figure 5C).

### 3.5. Association of Tumour Fucosylation with Cancer Hallmarks and Receptor Tyrosine Kinase Pathways

To define the fundamental pathway differences between fucosylation-high and fucosylation-low colorectal cancers, we performed comprehensive gene set enrichment analysis using MSigDB Hallmark and Reactome Pathway databases. This analysis revealed a striking dichotomy in cancer hallmark engagement between the two fucosylation-defined subsets, with each exhibiting mutually exclusive activation of distinct biological programmes that align perfectly with their histogenetic status, clinicopathological features, and clinical outcomes (Table S6: Supplementary Materials S6 and Table S7: Supplementary Materials S7).

#### 3.5.1. Hallmark Enrichment in Low-Fucosylation Tumours: The Invasive, Proliferative Phenotype

**Figure 6:**
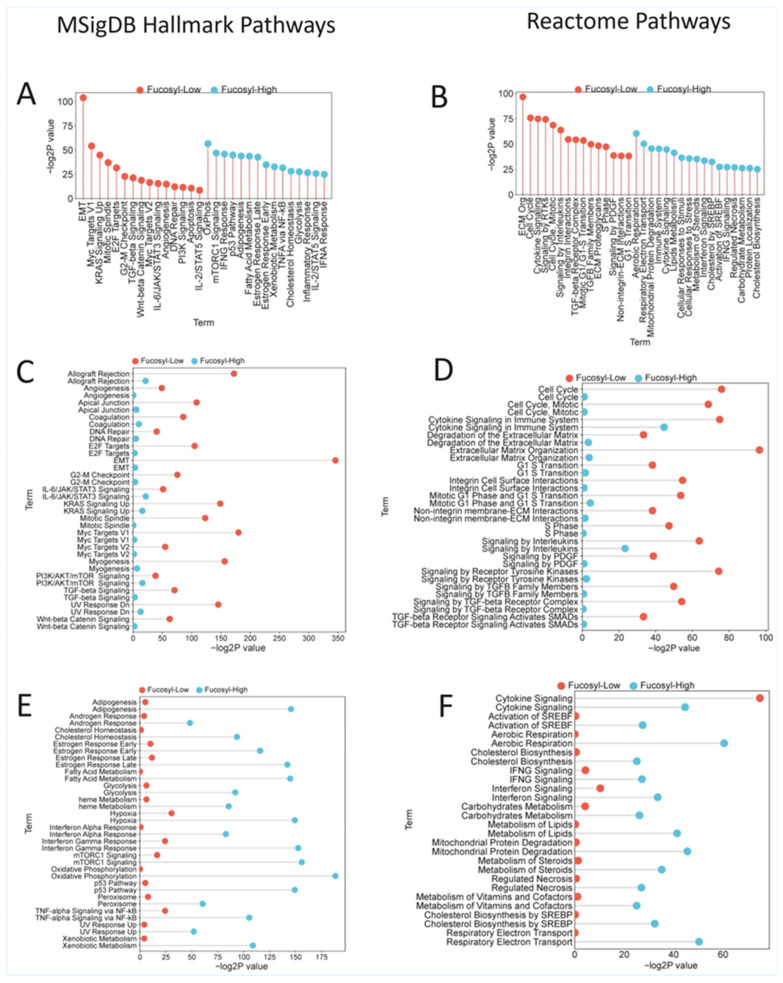
Differential pathway enrichment between fucosylation-defined colorectal cancer subsets. Enrichment analysis was performed using MSigDB Hallmark and Reactome pathway collections. (**A**,**C**,**E**) MSigDB Hallmark pathways; (**B**,**D**,**F**) Reactome pathways. (**A**,**B**) Top-ranked enriched pathways displayed as vertical lollipop plots (−log_2_(*p* value)). Colours indicate enrichment in fucosyl-low (red) or fucosyl-high (blue) tumours. (**C**,**D**) Pathways related to tumour signalling and microenvironmental interaction. Fucosyl-low tumours show enrichment of EMT, KRAS, TGF-β, WNT/β-catenin, PI3K–AKT–mTOR, IL6–JAK–STAT3, angiogenesis, E2F targets, and G2M checkpoint pathways. (**E**,**F**) Metabolic and stress-response pathways. Fucosyl-high tumours are enriched for oxidative phosphorylation, fatty acid metabolism, cholesterol homeostasis, glycolysis, xenobiotic metabolism, mTORC1 signalling, and interferon responses. Overall, fucosyl-low tumours preferentially engage invasion and proliferative programmes, whereas fucosyl-high tumours are characterised by metabolic and stress-adaptive pathways.

Low-fucosylation tumours demonstrated profound enrichment of the hallmarks associated with invasion, proliferation, and aggressive tumour behaviour (Table S6: Supplementary Materials S6, Figure 6A,B). The most significant enrichment was observed for activating invasion and metastasis, with epithelial–mesenchymal transition showing extraordinarily high significance (*p* = 9.21 × 10^−105^), accompanied by comprehensive activation of extracellular matrix organization, integrin signalling, and MET-mediated motility programmes. Sustaining proliferative signalling was highly significant, driven predominantly by Myc transcriptional programmes (*p* = 6.17 × 10^−55^) and E2F-mediated cell cycle activation (*p* = 2.15 × 10^−32^). Evading growth suppressors was dominated by TGF-beta signalling (*p* = 4.97 × 10^−22^), a pathway known to drive EMT and maintain stemness, with only modest p53 pathway enrichment (*p* = 0.028)—consistent with the high frequency of *TP53* mutations observed in this subset (Table S6: Supplementary Materials S6, Figure 6C,D). Genome instability hallmarks were significantly enriched, with emphasis on G2-M checkpoint control (*p* = 1.64 × 10^−23^) and DNA repair pathways, aligning with the chromosomal instability phenotype characteristic of low-fucosylation tumours. Inducing angiogenesis was highly significant (*p* = 1.67 × 10^−15^), indicating active vascular recruitment to support proliferation and invasion. Deregulating cellular metabolism was modest and restricted to glycolysis (*p* = 0.013), with no enrichment of oxidative phosphorylation or fatty acid metabolism—a glycolytic Warburg profile typical of undifferentiated, proliferative cancer cells. Resisting cell death showed moderate enrichment of apoptosis pathways (*p* = 2.25 × 10^−11^), though these were less prominent than the invasive and proliferative hallmarks (Table S6: Supplementary Materials S6, Figure 6C,D).

#### 3.5.2. Hallmark Enrichment in High-Fucosylation Tumours: The Metabolic, Differentiated, Non-Invasive Phenotype

High-fucosylation tumours exhibited a fundamentally different hallmark architecture, dominated by metabolic reprogramming and stress-response pathways, with the complete absence of invasion programmes (Table S6: Supplementary Materials S6, Figure 6A,B). Deregulating cellular metabolism emerged as the dominant hallmark, with extraordinary enrichment of oxidative phosphorylation (*p* = 2.10 × 10^−57^), fatty acid metabolism (*p* = 2.62 × 10^−44^), glycolysis (*p* = 2.32 × 10^−28^), and comprehensive metabolic reprogramming, encompassing the TCA cycle, mitochondrial beta-oxidation, respiratory electron transport, cholesterol biosynthesis, and peroxisomal pathways (Reactome: *p* = 1.78 × 10^−64^). This oxidative phosphorylation-dominant profile is characteristic of differentiated epithelial cells with functional mitochondria. Sustaining proliferative signalling was driven by mTORC1 (*p* = 1.18 × 10^−47^) and PI3K/AKT/mTOR pathways, but notably, Myc and E2F targets were completely absent, suggesting proliferation that is metabolically coupled rather than driven by classical oncogenic transcription factors. Resisting cell death was highly significant, dominated by p53 pathway activation (*p* = 1.22 × 10^−45^), apoptosis (*p* = 1.26 × 10^−22^), reactive oxygen species pathways, and comprehensive cell death programmes, including regulated necrosis and pyroptosis—consistent with functional tumour suppressor activity in differentiated cells (Table S6: Supplementary Materials S6, Figure 6E,F). Genome instability hallmarks were significantly enriched through p53-mediated DNA damage response and DNA repair pathways, indicating maintained genome surveillance. Evading growth suppressors was exclusively mediated by p53 pathways, with the complete absence of TGF-beta signalling. Inducing angiogenesis was limited to hypoxia response, with no enrichment of angiogenesis or VEGFR pathways. Critically, activating invasion and metastasis hallmarks were completely absent—EMT was not significant (*p* = 0.085), extracellular matrix organization showed no enrichment, and all invasion-related pathways failed to reach significance, providing pathway-level validation of their non-aggressive behaviour. Enabling replicative immortality showed only borderline significance, limited to mitotic G1 phase transition, with no enrichment of E2F targets, Myc targets, or G2-M checkpoint pathways, consistent with a quiescent or slow-cycling differentiated phenotype (Table S6: Supplementary Materials S6, Figure 6E,F).

#### 3.5.3. Receptor Tyrosine Kinase Pathways: Divergent Signalling Architectures

The RTK pathway analysis revealed fundamentally distinct signalling architectures that provide the upstream mechanistic explanation for these divergent hallmark patterns.

**Figure 7:**
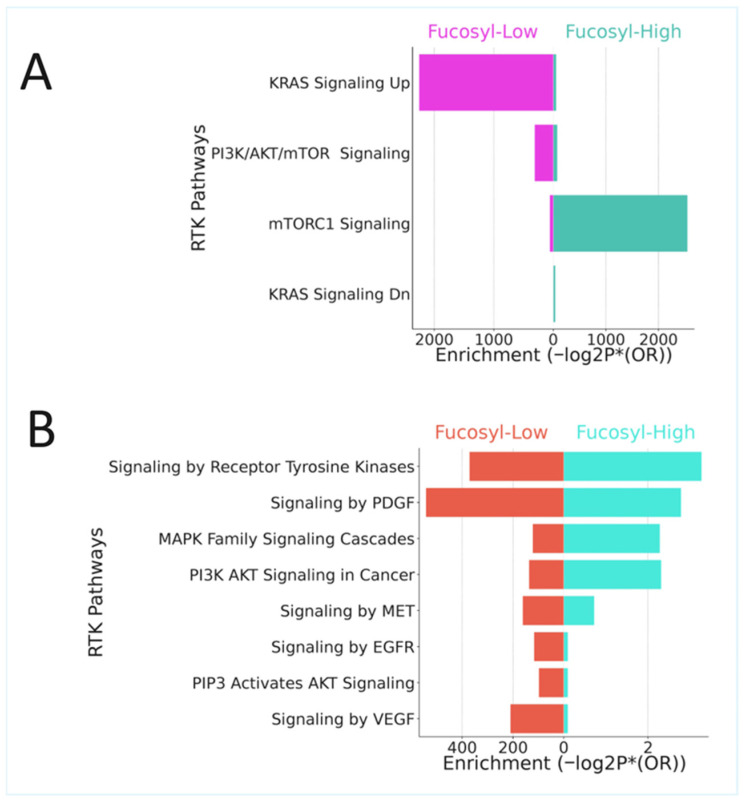
Differential enrichment of receptor tyrosine kinase (RTK) signalling pathways according to tumour fucosylation status in colorectal cancer. Bar plots show pathway enrichment comparing low-fucosylation and high-fucosylation colorectal cancer tumours. Enrichment scores are expressed as −log_2_(P) × odds ratio (OR), integrating statistical significance and effect size for pathway over-representation. (**A**) Hallmark RTK-related oncogenic pathways. Low-fucosylation tumours show strong enrichment for KRAS signalling and PI3K/AKT/mTOR signalling, whereas high-fucosylation tumours display prominent enrichment of mTORC1 signalling, indicating preferential activation of downstream mTOR pathway signalling in this subset. (**B**) Reactome RTK-related signalling pathways. Canonical RTK signalling programs—including signalling by receptor tyrosine kinases, PDGF signalling, MAPK family signalling cascades, MET signalling, and EGFR signalling—are significantly enriched in the low-fucosylation subset but do not reach statistical significance in the high-fucosylation subset. PI3K/AKT/mTOR signalling is enriched in both subsets, while mTORC1 signalling is strongly enriched in high-fucosylation tumours. Note that the *x*-axis scaling differs between panels to accommodate the large difference in enrichment magnitude between groups. In the Reactome RTK panel, enrichment scores for the low-fucosylation subset are substantially larger, necessitating differential scaling to allow visualization of the comparatively smaller enrichment values observed in the high-fucosylation subset.

Low-fucosylation tumours demonstrated comprehensive, multi-family RTK activation, with complete signalling cascades from receptor to effector. Signalling by receptor tyrosine kinases was highly significant (Reactome: *p* = 4.10 × 10^−23^), encompassing the EGFR (*p* = 1.90 × 10^−5^), PDGFR (*p* = 2.20 × 10^−12^), MET (*p* = 2.09 × 10^−7^), FGFR, IGFR, and VEGFR families. Downstream cascades included RAS-RAF-MEK-ERK signalling (*p* = 7.43 × 10^−10^) and PI3K-AKT pathways (*p* = 1.47 × 10^−7^) (Table S7: Supplementary Materials S7, Figure 7A,B). The gene composition revealed complete signalling complexes at every level: RTK receptors (EGFR, MET, PDGFRB, FGFR1, IGF1R), adaptor proteins (SHC1, GRB2, SOS1, GAB1), downstream kinases (RAF1, *BRAF*, MAP2K1/2, MAPK1/ERK2, MAPK3/ERK1, AKT1-3), transcription factors (MYC, FOS, JUN, ETV4, STAT1/3/5), and motility effectors (PTK2/FAK, MMP2/9, collagen family members) (Table S7: Supplementary Materials S7). This comprehensive architecture indicates that low-fucosylation tumours possess fully intact RTK signalling systems capable of receiving diverse extracellular inputs and translating them into coordinated proliferative, invasive, and angiogenic outputs. In striking contrast, high-fucosylation tumours exhibited the complete absence of RTK pathway enrichment at the receptor level. Signalling by receptor tyrosine kinases was not significant (*p* = 0.21), with no enrichment of any RTK family. Adaptor proteins and classical downstream effectors, including RAS, RAF, and ERK, were notably absent. Instead, high-fucosylation tumours demonstrated selective activation of downstream metabolic modules without upstream RTK engagement. mTORC1 signalling dominated (*p* = 1.18 × 10^−47^), with PI3K-AKT pathway components present but appearing to function in nutrient sensing rather than RTK-driven contexts. MAPK signalling was limited to stress-activated p38, and JAK-STAT was minimal, with only STAT3 enrichment. Gene composition reflected metabolic regulation (LDHA, HK2, GOT1, IDH1, PFKFB3), stress response (ATF4, DDIT4, SESN2), and autophagy (ULK1, SQSTM1), with the complete absence of invasion effectors, including MMPs, collagens, integrins, and FAK (Table S7: Supplementary Materials S7). This architecture indicates that high-fucosylation tumours completely lost RTK signalling at the receptor level, retaining only selective downstream modules that function in nutrient sensing, stress response, and metabolic regulation (Table S7: Supplementary Materials S7, Figure 7).

#### 3.5.4. The Differentiation Connection Unifies the Pathway Architecture

The information that high-fucosylation tumours have a higher epithelial differentiation index provides the unifying framework that explains these opposing pathway architectures. Differentiated epithelial cells characteristically downregulate RTK expression as they exit the stem/progenitor state, shift from RTK-driven proliferation to metabolic homeostasis, engage nutrient-sensing pathways rather than growth factor pathways, and respond to stress through p53 and autophagy rather than invasion. Conversely, low-fucosylation tumours represent the undifferentiated, stem-like state, wherein RTKs are highly expressed, complete signalling cascades drive proliferation and survival, TGF-beta cooperates with RTKs to induce EMT, and invasion effectors are transcriptionally active (Figure 6, Table S6: Supplementary Materials S6, Figure 7, Table S7: Supplementary Materials S7).

## 4. Discussion

**Figure 8:**
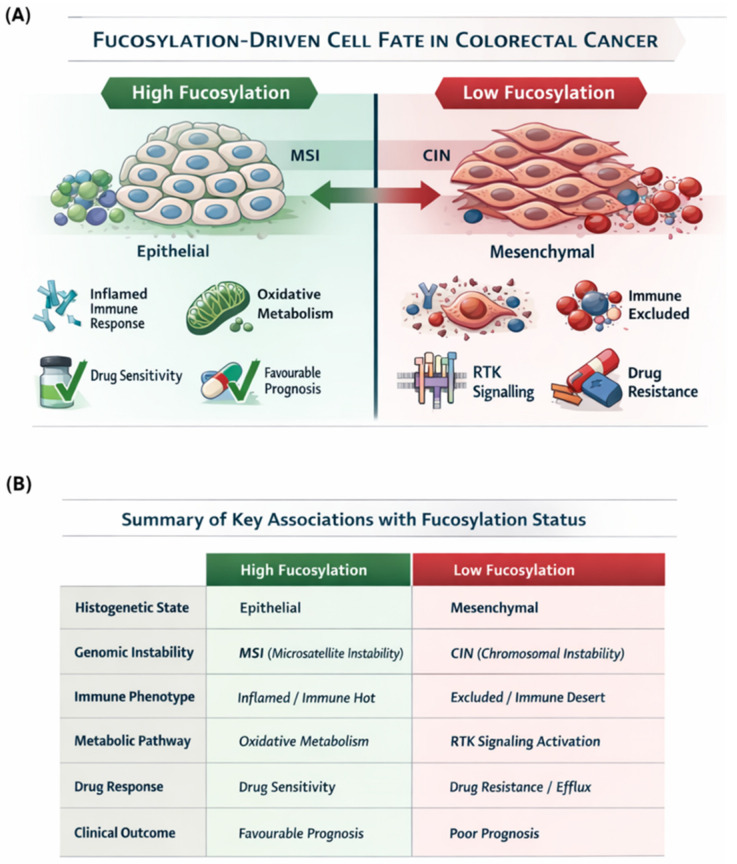
Fucosylation-driven cell fate and associated molecular features in colorectal cancer. (**A**) Schematic representation of the biological consequences of differential fucosylation in colorectal cancer. High fucosylation is associated with an epithelial phenotype and microsatellite instability (MSI), characterized by an inflamed/immune-active tumor microenvironment, enhanced oxidative metabolism, increased drug sensitivity, and favourable clinical prognosis. In contrast, low fucosylation is linked to a mesenchymal phenotype and chromosomal instability (CIN), accompanied by immune exclusion, activation of receptor tyrosine kinase (RTK) signalling pathways, drug resistance, and poor clinical outcomes. The bidirectional arrow indicates phenotypic plasticity between these states. (**B**) Tabulated summary of key clinicopathological and molecular associations with fucosylation status. High fucosylation correlates with epithelial differentiation, MSI, immune-inflamed phenotype, oxidative metabolic pathways, drug sensitivity, and favourable prognosis. Low fucosylation is associated with mesenchymal features, CIN, immune-desert/excluded phenotype, RTK pathway activation, drug resistance/efflux mechanisms, and poor prognosis.

The integrative analysis presented in this study positions tumour fucosylation as a critical determinant of cancer cell fate in colorectal carcinoma, unifying histogenetic status, genomic architecture, microenvironmental remodelling, therapeutic resistance, and clinical outcome into a coherent biological framework. These findings are summarised in an integrative framework (Figure 8), highlighting tumour fucosylation as a central determinant of colorectal cancer cell fate. Our findings demonstrate that fucosylation status defines two fundamentally distinct tumour cell states that align with the hypermutated MSI pathway and the chromosomal instability pathway, respectively, and that these states exhibit the mutually exclusive engagement of cancer hallmarks, RTK signalling architectures, and multidrug resistance programmes. These observations have significant implications for understanding CRC heterogeneity, risk stratification, and therapeutic decision-making.

The strong association between high fucosylation and elevated epithelial differentiation index across multiple independent metrics establishes tumour fucosylation as a robust correlate of histogenetic status. This finding aligns with emerging evidence that glycosylation programmes are intimately linked to cellular differentiation states. Recent work by Bastian and colleagues demonstrated that core fucosylation via FUT8 is dynamically regulated during intestinal epithelial differentiation, with differentiated enterocytes exhibiting distinct fucosylation patterns compared to stem or progenitor cells [5]. The reciprocal relationship between fucosylation and differentiation observed in our study—wherein high fucosylation marks well-differentiated tumours and low fucosylation marks poorly differentiated, mesenchymal tumours—suggests that fucosylation may actively participate in maintaining the differentiated epithelial phenotype rather than merely reflecting it.

This interpretation is supported by mechanistic studies demonstrating that specific fucosyltransferases directly regulate epithelial homeostasis. Moriwaki and colleagues showed that GMDS deficiency, which ablates cellular fucosylation, renders colon cancer cells resistant to death receptor-mediated apoptosis and promotes metastatic potential, indicating that intact fucosylation programmes are required for maintaining normal epithelial cell behaviour [23]. Core fucosylation mediated by FUT8 has been shown to modulate the stability and function of cell-surface receptors, including the Wnt co-receptor LRP6, where in situ fucosylation influences receptor endocytosis and Wnt/β-catenin signalling [24]. These findings suggest that fucosylation is not merely a passive marker of differentiation but can actively regulate signalling networks that govern epithelial identity and stemness in intestinal epithelial cells.

The relationship between fucosylation and differentiation extends beyond the intestinal epithelium. In hepatocellular carcinoma, FUT8 expression has been associated with poorly differentiated and more aggressive tumour phenotypes [25]. Similarly, in prostate cancer, FUT8 overexpression correlates with higher Gleason grade, a histological indicator of reduced differentiation [26]. These observations across multiple cancer types suggest that the association between fucosylation and differentiation represents a conserved biological principle with broad relevance to epithelial cancers.

The striking dichotomy in genomic features between fucosylation subsets—with high-fucosylation tumours enriched for *BRAF* mutations, MSI-H status, and hypermutated phenotype and low-fucosylation tumours characterised by *TP53* mutations, MSS status, and chromosomal instability—provides a molecular foundation for the observed phenotypic differences. These findings align with the established paradigm that CRC comprises distinct molecular subtypes with different clinical behaviours and therapeutic vulnerabilities [27]. To further resolve the molecular identity of fucosylation-defined subsets, we examined their distribution across established colorectal cancer molecular subtypes [27]. The enrichment of high-fucosylation tumours in the Hypermutated/MSI molecular subtype and low-fucosylation tumours in Epithelial/CIN and Mesenchymal/EMT/GS subtypes indicates that fucosylation status captures the fundamental molecular dichotomy in CRC pathogenesis.

Recent advances in understanding the regulation of fucosyltransferases in cancer provide mechanistic insight into these associations. A comprehensive review by Keeley and colleagues highlights that FUT8 expression is modulated by multiple transcription factors and non-coding RNAs in a tissue-specific manner, with p53 emerging as a key transcriptional regulator of FUT8 in colorectal and hepatocellular carcinoma [16]. The strong p53 pathway enrichment observed in high-fucosylation tumours, coupled with the relative absence of *TP53* mutations in this subset, suggests that functional p53 may promote fucosylation programmes that reinforce epithelial differentiation. Conversely, the enrichment of *TP53* mutations in low-fucosylation tumours may contribute to the loss of fucosylation and subsequent adoption of mesenchymal phenotypes. Supporting this, Okagawa and colleagues demonstrated that p53 directly binds to the FUT8 promoter and regulates its expression, with p53 knockdown leading to decreased FUT8 levels in cancer cells [28]. This provides direct evidence that p53 status influences core fucosylation capacity.

The association between high fucosylation and the MSI-H/*BRAF*-mutant pathway is particularly intriguing. This strong association between high fucosylation and MSI status provides a molecular explanation for the favourable prognosis observed in high-fucosylation tumours, as MSI-H colorectal cancers are well-established to have better stage-adjusted survival compared to MSS tumours and demonstrate distinct responses to immunotherapy [29,30]. Independent glycomic analyses of MSI CRC tissues have reported distinct differences in the abundances of multiple fucosylated N glycan classes between MSI and MSS samples, including elevated fucosylated pauci mannose and hybrid structures in MSI tumours [31]. Moreover, fucosyltransferases—the enzymes responsible for generating terminal fucosylated epitopes such as Lewis and sialyl Lewis antigens—are dysregulated in CRC, and their products have been implicated in tumour progression, metastatic potential, and interactions with selectins and immune components [32]. Together, these findings support the idea that distinct fucosylation signatures may both mark and mechanistically contribute to differences in tumour behaviour and immune recognition in MSI H colorectal cancer. Notably, oncogenic *BRAF* V600E has been shown to regulate glycosyltransferase expression through MAPK-dependent signalling, including the upregulation of GALNT3 in colorectal cancer cells, thereby altering tumour glycosylation programmes [33]. Although this study examined O-linked glycosylation rather than fucosylation specifically, it supports the broader concept that oncogenic *BRAF* signalling can reprogram glycosylation pathways in colorectal cancer.

Perhaps the most striking finding of our study is the complete divergence in RTK signalling architecture between fucosylation subsets. Low-fucosylation tumours exhibit comprehensive, multi-family RTK activation, with complete signalling cascades from receptor to effector, encompassing EGFR, PDGFR, MET, FGFR, IGFR, and VEGFR families, with downstream engagement of RAS-RAF-MEK-ERK and PI3K-AKT pathways. In contrast, high-fucosylation tumours show the complete absence of RTK pathway enrichment at the receptor level, retaining only downstream metabolic modules functioning in nutrient sensing and stress response.

This dichotomy has profound implications for understanding how fucosylation status governs cellular responsiveness to microenvironmental cues. The fucosylation of RTKs themselves may play a critical role in their signalling competence. Li and colleagues recently demonstrated that the FUT8-mediated core fucosylation of EGFR is essential for receptor dimerization and autophosphorylation, with the loss of fucosylation resulting in attenuated downstream signalling and reduced proliferative capacity [34]. Similarly, Tu and colleagues showed that MET receptor fucosylation by FUT8 regulates HGF-induced cell migration and invasion, providing a direct mechanistic link between fucosylation status and invasive potential [35]. The complete absence of RTK signalling in high-fucosylation tumours may therefore reflect not only the downregulation of receptor expression but also loss of the glycosylation-dependent modifications required for receptor function.

Beyond direct receptor fucosylation, the glycan microenvironment created by tumour cells can influence RTK signalling through the modulation of ligand availability and receptor clustering. As reviewed by Gao and colleagues, glycans on key cell surface receptors, including RTKs, play critical roles in regulating receptor conformation, dimerization, and signal amplification, thereby directly impacting cell proliferation and survival [36]. The loss of such glycan-dependent regulatory mechanisms in low-fucosylation tumours may contribute to the aberrant RTK signalling observed in this subset.

The selective retention of mTORC1 signalling in high-fucosylation tumours, despite the absence of upstream RTK input, indicates that these tumours maintain nutrient-sensing and metabolic regulatory capacity, independent of growth factor signalling. This finding aligns with recent work by Mossmann and colleagues demonstrating that mTORC1 can be activated by amino acid availability through the Rag GTPase pathway, independently of RTK-PI3K signalling, allowing cells to coordinate metabolism with nutrient status [37]. In well-differentiated epithelial cells, this RTK-independent mTORC1 activation likely supports homeostatic metabolic functions rather than proliferative growth. Experimental studies have shown that mTORC1 signalling directly regulates intestinal epithelial differentiation, with activation of mTORC1 altering goblet and Paneth cell lineages and epithelial homeostasis, supporting a functional role in differentiated epithelial states rather than proliferation-dominant programs [38].

The metabolic dichotomy between fucosylation subsets—with low-fucosylation tumours exhibiting glycolytic metabolism and high-fucosylation tumours demonstrating dominant oxidative phosphorylation with comprehensive metabolic reprogramming—provides another layer of biological distinction. This pattern aligns with the emerging understanding that cellular differentiation state is tightly coupled to metabolic programmes [39]. Differentiated epithelial cells characteristically utilise oxidative phosphorylation to meet their energetic demands, while undifferentiated, proliferative cells adopt glycolytic metabolism even under aerobic conditions—the Warburg effect.

Recent studies have identified links between fucosylation and metabolic regulation. Core fucosylation mediated by FUT8 regulates receptor signalling pathways such as EGFR, which are known to control cellular metabolic programmes. Activation of EGFR signalling promotes glycolysis through PI3K–AKT–dependent stabilisation of HIF-1α, leading to transcriptional upregulation of glucose transporter 1 (GLUT1) and enhanced glucose uptake. This provides a mechanistic framework linking fucosylation to glycolytic flux. More broadly, glycosylation is tightly coupled to cellular metabolism, as glycan biosynthesis depends on metabolic flux and nucleotide sugar availability [40]. In high-fucosylation tumours, the enrichment of oxidative phosphorylation pathways suggests that these tumours maintain functional mitochondria and utilise oxidative metabolism, consistent with their differentiated phenotype. Conversely, the glycolytic profile of low-fucosylation tumours aligns with their proliferative, undifferentiated state and may contribute to their therapeutic resistance, as glycolytic tumours are known to be less responsive to various chemotherapeutic agents [41].

The comprehensive metabolic reprogramming observed in high-fucosylation tumours extends beyond oxidative phosphorylation to encompass fatty acid metabolism, cholesterol biosynthesis, and peroxisomal pathways. This metabolic profile is characteristic of differentiated epithelial cells that require lipid synthesis for membrane maintenance and specialised functions. Seminal work by Cheng and colleagues demonstrated that N-glycosylation of the SREBP cleavage-activating protein (SCAP) is essential for its stability, trafficking to the Golgi, and subsequent activation of SREBP-1, a master transcription factor controlling lipid biosynthesis genes [42]. This establishes a direct mechanistic link between glycosylation status and lipid metabolic programming. The absence of such glycosylation-dependent SCAP regulation in low-fucosylation tumours may contribute to their reliance on glycolytic metabolism and their inability to engage oxidative and lipid anabolic programmes.

The enrichment of peroxisomal pathways in high-fucosylation tumours is particularly noteworthy. Peroxisomes play critical roles in lipid metabolism and reactive oxygen species homeostasis, both of which are central to tumour biology and cellular transformation. Beyond these functions, peroxisomal activity has been increasingly linked to the regulation of cellular metabolic state and plasticity. In this context, the enrichment of peroxisomal pathways in high-fucosylation tumours suggests a metabolic profile consistent with preserved mitochondrial function and differentiated epithelial states. Conversely, reduced peroxisomal activity may contribute to metabolic reprogramming, oxidative stress imbalance, and the emergence of more proliferative, less differentiated tumour phenotypes [43]. The presence of intact peroxisomal programmes in high-fucosylation tumours may therefore contribute to their maintenance of epithelial differentiation and their resistance to mesenchymal transformation.

The enrichment of immune-excluded and immune-desert phenotypes in low-fucosylation tumours, coupled with the complete absence of such immunosuppressive states in high-fucosylation tumours, provides important insights into the relationship between fucosylation and antitumour immunity. These findings are consistent with the established concept that mesenchymal, EMT-high tumours are associated with immune exclusion and resistance to immunotherapy [44]. The lack of association between fucosylation status and Siglec signalling, despite the established role of fucosylated glycans as Siglec ligands, suggests that the immune-evasive phenotype of low-fucosylation tumours is mediated through EMT-associated mechanisms rather than direct glycan-immune receptor interactions.

Recent advances in understanding the glyco-immune checkpoint have highlighted the complexity of glycosylation in regulating antitumour immunity. Rodríguez and colleagues demonstrated that tumour-associated glycans, including fucosylated structures, can engage inhibitory Siglec receptors on immune cells, contributing to immune evasion [45]. However, our findings suggest that in CRC, the dominant mechanism of immune evasion in low-fucosylation tumours is likely the physical exclusion of immune cells by the desmoplastic stroma and the immunomodulatory effects of EMT programmes, rather than direct Siglec engagement. This interpretation is supported by the strong enrichment of stromal and ECM remodelling pathways in low-fucosylation tumours, which would be expected to create physical barriers to immune infiltration [46].

The relationship between fucosylation and immune recognition may be context-dependent. In MSI-H tumours, which are enriched in the high-fucosylation subset, the high mutation burden generates neoantigens that drive robust T-cell responses. The differentiated, epithelial phenotype of these tumours may facilitate immune infiltration, whereas the desmoplastic stroma of low-fucosylation tumours may impede it. De Vries and colleagues recently demonstrated that EMT-associated ECM remodelling creates a physical barrier to T-cell infiltration and that targeting the stromal microenvironment can enhance immunotherapy efficacy [47]. Our findings suggest that fucosylation status could identify the patients most likely to benefit from such stroma-targeting approaches.

The absence of Siglec signalling enrichment in either subset, despite the established role of fucosylated glycans as Siglec ligands, warrants comment. It is possible that Siglec engagement is regulated at the post-transcriptional level or that the relevant Siglec ligands are not captured by transcriptomic analysis. Alternatively, as suggested by van de Wall and colleagues, the immune-evasive effects of fucosylation may be mediated through other glycan-binding receptors, such as the selectins or galectins, which were not specifically evaluated in our analysis [48].

The striking dichotomy in multidrug resistance mechanism engagement between fucosylation subsets has important therapeutic implications. Low-fucosylation tumours preferentially activate drug efflux, a classical resistance mechanism involving the active extrusion of chemotherapeutic agents. This finding aligns with their aggressive biology, mesenchymal phenotype, and poor prognosis and suggests that these tumours may be intrinsically resistant to conventional chemotherapy through physical elimination of drugs from the intracellular compartment. Recent studies have demonstrated that EMT transcription factors directly upregulate ABC transporter expression, providing a mechanistic link between the mesenchymal phenotype observed in low-fucosylation tumours and their enhanced drug efflux capacity [49].

Conversely, high-fucosylation tumours preferentially engage a coordinated programme of xenobiotic sensing, target bypass signalling, and drug trafficking and sequestration. This more sophisticated resistance strategy, focused on drug recognition, adaptive survival pathway activation, and subcellular drug compartmentalisation, coexists with their otherwise favourable tumour biology. The enrichment of these mechanisms in high-fucosylation tumours suggests that while these tumours are generally indolent and treatment-responsive, they possess an intrinsic adaptive capacity that could be unmasked by targeted therapeutic challenge. This finding aligns with recent review-based observations indicating that MSI-H colorectal cancers, despite their generally favourable prognosis, can exhibit mechanisms of acquired or adaptive resistance to immune checkpoint blockade through alternative immune evasion pathways [50].

The mutual exclusivity of these resistance programmes—with a drug efflux unique to low-fucosylation tumours and the xenobiotic sensing-target bypass-sequestration triad unique to high-fucosylation tumours—indicates that fucosylation status captures fundamental differences in how tumours adapt to and survive therapeutic challenge. This observation aligns with the emerging concept that drug resistance mechanisms are not randomly distributed but are linked to specific tumour cell states and differentiation programmes [51].

The enrichment of xenobiotic sensing pathways in high fucosylation tumours is particularly intriguing. These pathways, which include nuclear receptors such as the pregnane X receptor (PXR) and the constitutive androstane receptor (CAR) as well as phase I/II drug metabolizing enzymes, enable cells to detect and respond to foreign compounds by regulating the expression of detoxification enzymes and transporters. PXR and CAR are highly expressed not only in the liver but also in the intestinal epithelium, where they modulate the transcription of xenobiotic metabolism genes and interact with the gut microbiome to influence host responses to environmental chemicals and gut-derived metabolites [52]. The presence of these pathways in high fucosylation tumours may therefore reflect the retention of differentiated epithelial functions, including the capacity to sense and respond to dietary compounds, xenobiotics, and chemotherapeutic agents.

The drug trafficking and sequestration mechanisms enriched in high-fucosylation tumours involve the lysosomal and endosomal compartmentalisation of chemotherapeutic agents. This resistance mechanism is particularly relevant to weakly basic drugs such as doxorubicin and sunitinib, which accumulate in acidic lysosomes. Zhitomirsky and Assaraf demonstrated that lysosomal sequestration is an active, energy-dependent process that can be targeted pharmacologically [19]. The enrichment of this mechanism in high-fucosylation tumours suggests that these patients might benefit from combination regimens, including lysosomotropic agents or inhibitors of lysosomal function.

The comprehensive hallmark analysis reveals that fucosylation status integrates multiple cancer hallmarks into coherent phenotypic programmes. Low-fucosylation tumours engage the canonical hallmarks of aggressive cancer: sustaining proliferative signalling through Myc/E2F, evading growth suppressors through TGF-beta, activating invasion and metastasis through EMT, inducing angiogenesis, and resisting cell death through modest p53 engagement. This profile aligns with the classical understanding of cancer as a disease of uncontrolled proliferation and tissue invasion [53].

High-fucosylation tumours, in contrast, exhibit a fundamentally different hallmark architecture that more closely resembles normal differentiated tissue than aggressive cancer. The dominance of metabolic reprogramming, particularly oxidative phosphorylation, coupled with strong p53-mediated stress responses and the complete absence of invasion programmes, suggests that these tumours retain elements of tumour suppressor function and tissue homeostasis. This profile may explain their excellent prognosis and may indicate that they are less “transformed” in the classical sense, despite being malignant.

The complete absence of invasion and metastasis hallmarks in high-fucosylation tumours is particularly striking and has important clinical implications. These tumours, despite their malignant potential, appear to lack the machinery required for tissue invasion and metastasis. This finding aligns with the concept of “indolent” cancers that may never progress to clinically significant disease—a phenomenon well-documented in prostate and thyroid cancer but less recognised in CRC. Esserman and colleagues have championed the concept of indolent lesions with low malignant potential, arguing that such lesions may be overtreated [54]. Our findings suggest that high fucosylation could serve as a biomarker to identify CRC patients with indolent disease who might be candidates for less intensive management.

The demonstration that fucosylation status independently predicts overall and disease-free survival, with high-fucosylation patients exhibiting nearly double the median survival of low-fucosylation patients, positions tumour fucosylation as a robust prognostic biomarker in colorectal cancer. The independence of this effect from tumour stage and patient age, confirmed by multivariable Cox regression, indicates that fucosylation status captures a biological heterogeneity that is not fully reflected by conventional clinicopathological parameters. The dose–response relationship observed with continuous fucosylation scores further validates fucosylation as a biologically relevant continuous variable, not merely a categorical classifier.

These findings have immediate translational potential. Fucosylation status could be incorporated into existing molecular classification systems to refine prognostic stratification and guide treatment decisions. For example, low-fucosylation tumours, with their aggressive biology, comprehensive RTK signalling, and drug efflux-mediated resistance, may benefit from intensive follow-up and combination chemotherapy regimens that include P-glycoprotein inhibitors or agents that are not efflux pump substrates. The potential of repurposing existing FDA-approved drugs with P-glycoprotein inhibitory activities, as reviewed by Lai and colleagues, could be evaluated in this context to overcome efflux-mediated resistance [55].

Conversely, high-fucosylation tumours, despite their favourable prognosis, harbour adaptive resistance mechanisms that may be unmasked by targeted therapies, suggesting that these patients could benefit from upfront combination strategies to prevent target bypass–mediated resistance. The target bypass signalling pathways enriched in these tumours—including alternative RTK activation and downstream pathway redundancy—may be effectively addressed through rational combinatorial approaches. For instance, adaptive resistance mediated by pathway reactivation and compensatory signalling has been shown to be overcome by concurrent inhibition of parallel pathways or multiple receptor tyrosine kinases in preclinical models [56]. The emergence of glycosylation-based therapeutics adds further translational relevance to our findings. Inhibitors of fucosylation, such as 2-fluorofucose, have shown promise in preclinical models by disrupting glycan-dependent signalling and sensitising tumours to chemotherapy and immunotherapy [57]. Our results suggest that such approaches may be particularly effective in low-fucosylation tumours, where restoration of fucosylation could potentially reverse mesenchymal phenotypes and attenuate RTK signalling. Conversely, high-fucosylation tumours may be more amenable to strategies targeting the adaptive resistance mechanisms we have identified, such as lysosomal sequestration inhibitors or agents that block xenobiotic-sensing pathways.

The distinct immune phenotypes of fucosylation subsets have implications for immunotherapy. Low-fucosylation tumours, with their immune-excluded and immune-desert microenvironments, are unlikely to respond to immune checkpoint blockade as monotherapy. However, combination approaches that target the desmoplastic stroma or reverse EMT could potentially render these tumours immunoresponsive. Mariathasan and colleagues demonstrated that TGF-beta signalling in the stroma creates an immune-excluded phenotype that limits checkpoint inhibitor efficacy, and that TGF-beta inhibition can overcome this resistance [58]. Given the strong TGF-beta enrichment in low-fucosylation tumours, this combination approach may be particularly relevant for this subset.

High-fucosylation tumours, with their MSI-H-enriched profile and absence of immune exclusion, are likely to be immunoresponsive. MSI-H tumours have demonstrated remarkable responses to immune checkpoint blockade across multiple studies [29,30]. However, as noted in Williams et al., even MSI-H tumours may develop resistance through mechanisms such as loss of antigen presentation or activation of alternative immune checkpoints [50]. The adaptive resistance mechanisms we identified in high-fucosylation tumours—target bypass signalling and drug sequestration—may also contribute to immunotherapy resistance and could be targeted pharmacologically.

Several limitations of this study should be acknowledged. First, although we integrated three independent cohorts (TCGA, CPTAC2, and Sidra–LUMC) to increase statistical power and generalisability, the use of multi-cohort transcriptomic data introduces the potential for residual confounding due to cohort-specific differences in patient characteristics, sequencing platforms, and data processing pipelines. While ComBat batch correction was applied and quantitatively validated, with batch-associated variance reduced from R^2^ = 0.85 before correction to approximately R^2^ ≈ 0 after correction based on PCA coordinates, subtle residual confounding cannot be completely excluded, particularly for higher-order transcriptomic features not captured by the principal components.

Second, the observational and retrospective design of this study precludes causal inference. While we identified strong associations between fucosylation status and multiple tumour phenotypes, these findings remain correlative and hypothesis-generating. Functional studies using in vitro and in vivo models are required to determine whether manipulating fucosylation directly alters tumour cell state and therapeutic response. Such studies could include CRISPR-mediated perturbation of fucosyltransferases or pharmacological inhibition of fucosylation to assess effects on differentiation, RTK signalling, metabolism, immune evasion, and drug resistance.

Third, our analysis was performed at the transcriptomic level, which may not fully capture the structural and functional complexity of glycans. Proteomic and glycomic approaches, including mass spectrometry-based glycan profiling, would be required to directly quantify fucosylated proteins and glycan structures and to identify the specific molecular mediators underlying the observed phenotypes [59].

Fourth, the study lacks functional clinical endpoints, including treatment response, performance status, and quality-of-life measures. Consequently, the observed associations between fucosylation status and clinical outcomes should be interpreted as prognostic rather than predictive, and their relevance to therapeutic decision making requires prospective evaluation.

Fifth, reliance on publicly available datasets introduces inherent limitations, including potential coding inconsistencies, missing variables, and heterogeneity in clinical annotation and treatment regimens, which were not standardised across cohorts. These factors may contribute to unmeasured variability that cannot be fully controlled.

Sixth, although the integration of three independent cohorts provides internal robustness, formal external validation in a completely independent dataset was not performed. Future studies should validate the fucosylation signature in independent cohorts and assess its clinical utility in prospective, uniformly treated patient populations.

Seventh, this study did not distinguish between different forms of fucosylation, such as core versus O-fucosylation, which are mediated by distinct fucosyltransferases and may have non-overlapping biological functions [60]. Future work should examine the specific contributions of individual fucosylation pathways to tumour phenotypes.

Eighth, the analysis was conducted on bulk tumour transcriptomic data, which averages signals across malignant and stromal compartments. Single-cell approaches would be valuable to determine whether the observed fucosylation signature reflects cell-intrinsic programmes or tumour microenvironmental composition, and to resolve intratumoural heterogeneity [61].

Finally, although distinct multidrug resistance programmes were identified across fucosylation-defined subsets, functional validation of these mechanisms is required. Experimental models such as patient-derived organoids or xenografts stratified by fucosylation status could be used to evaluate therapeutic responses and test strategies to overcome fucosylation-associated resistance mechanisms. Future research should focus on several key areas. First, elucidating the mechanistic links between fucosylation and the phenotypic features we have identified will require detailed molecular studies. Questions to address include: Which specific fucosyltransferases are responsible for maintaining epithelial differentiation? How does fucosylation regulate RTK signalling at the molecular level? What is the relationship between fucosylation and metabolic reprogramming? How do fucosylation programmes interact with p53 and TGF-beta signalling pathways?

Second, preclinical studies evaluating fucosylation-based therapeutic strategies in patient-derived xenograft models stratified by fucosylation status could provide proof-of-concept for personalised approaches. Such studies could test: (i) fucosylation inhibitors in low-fucosylation tumours to determine whether restoring fucosylation reverses mesenchymal phenotypes; (ii) efflux pump inhibitors in low-fucosylation tumours to overcome chemotherapy resistance; (iii) lysosomal sequestration inhibitors in high-fucosylation tumours to block drug compartmentalisation; and (iv) combination strategies targeting the specific resistance mechanisms identified in each subset.

Third, the serial assessment of fucosylation signatures in liquid biopsies, leveraging the emerging potential of glycomarkers in translational medicine [62], could provide real-time information about tumour evolution and emerging resistance.

Fourth, the development of clinical-grade assays for fucosylation status will be essential for translation. Immunohistochemical approaches using antibodies specific for glycosylated epitopes could enable the rapid assessment of tumour glycosylation status in formalin-fixed, paraffin-embedded tissues [25]. Alternatively, transcriptomic-based classifiers could be developed for use with clinical gene expression platforms.

Finally, prospective clinical trials stratifying patients by fucosylation status could evaluate whether this biomarker improves treatment selection and patient outcomes. For example, a trial could randomise patients with low-fucosylation tumours to standard chemotherapy versus chemotherapy plus an efflux pump inhibitor, while patients with high-fucosylation tumours could be randomised to standard therapy versus therapy plus a lysosomal sequestration inhibitor. Such biomarker-driven trials represent the future of precision oncology.

## 5. Conclusions

This study establishes tumour fucosylation as an integrative determinant of cancer cell fate in colorectal carcinoma, defining two fundamentally distinct tumour cell states with mutually exclusive engagement of invasion programmes, RTK signalling architectures, metabolic pathways, immune phenotypes, and drug resistance mechanisms. High-fucosylation tumours correspond to the differentiated, hypermutated MSI pathway, characterised by oxidative metabolism, functional p53, absence of invasion machinery, xenobiotic sensing, target bypass signalling, drug sequestration, and excellent prognosis. Low-fucosylation tumours correspond to the undifferentiated chromosomal instability pathway, characterised by comprehensive multi-family RTK signalling, glycolytic metabolism, EMT, TGF-beta activation, immune exclusion, drug efflux-mediated resistance, and poor prognosis.

The independent prognostic value of fucosylation status, coupled with its integration of diverse biological programmes, positions it as a powerful biomarker for risk stratification and personalised treatment decisions in colorectal cancer. The dose–response relationship between continuous fucosylation scores and survival outcomes suggests that fucosylation is not merely a categorical classifier but a biologically relevant continuous variable that captures the degree of epithelial differentiation and the activity of underlying oncogenic programmes.

The mutual exclusivity of the biological programmes we identified—comprehensive RTK signalling and invasion in low-fucosylation tumours versus oxidative metabolism and p53 activity in high-fucosylation tumours—positions fucosylation as a transcriptomic readout of a fundamental binary cell fate decision between maintaining an undifferentiated, RTK-competent, proliferative state and adopting a differentiated, metabolically active, quiescent state. This decision has profound implications for tumour behaviour, therapeutic response, and patient outcome.

These findings open new avenues for therapeutic intervention targeting fucosylation-dependent signalling and resistance mechanisms. For low-fucosylation tumours, strategies could include fucosylation restoration to reverse mesenchymal phenotypes, efflux pump inhibition to overcome chemotherapy resistance, and TGF-beta blockade to alleviate immune exclusion. For high-fucosylation tumours, approaches could include targeting adaptive resistance mechanisms such as lysosomal sequestration, preventing target bypass through rational combination therapy, and leveraging their immunoresponsive MSI-H phenotype for immunotherapy. The integration of fucosylation status into clinical decision making has the potential to improve outcomes across the spectrum of colorectal cancer by matching patients with the therapies most likely to be effective based on the fundamental biology of their tumours.

## Figures and Tables

**Table 1:** Associations between fucosylation and immune/emt/stromal/siglec phenotypes.

Variable	Mann–Whitney U	Standardized Test Statistic	*p*-Value	FDR q-Value	Direction of Association
Immune_Inflamed	92,221	0.182	0.855	0.855	No significant difference
Immune_Excluded	74,204	−4.457	8.32 × 10^−6^	1.66 × 10^−5^	Higher in Low Fucosylation
Immune_Desert	65,759	−6.631	3.33 × 10^−11^	1.67 × 10^−10^	Higher in Low Fucosylation
EMT_score	73,966	−4.518	6.24 × 10^−6^	1.56 × 10^−5^	Higher in Low Fucosylation
Proliferation_score	86,065	−1.403	0.161	0.201	No significant difference
Stroma_score	70,353	−5.448	5.09 × 10^−8^	2.55 × 10^−7^	Higher in Low Fucosylation
EMT_stroma_score	72,091	−5.001	5.71 × 10^−7^	1.90 × 10^−6^	Higher in Low Fucosylation
EMT_proliferation_diff_score	81,796	−2.502	0.012	0.02	Higher in Low Fucosylation
Siglec_Score	85,714	−1.493	0.135	0.15	No significant difference

## Data Availability

All the data explored in this study have been deposited in the Genome Data Commons (https://portal.gdc.cancer.gov/) and the cBioPortal for Cancer Genome (https://www.cbioportal.org/) databases.

## Supplementary Materials

The following supporting information can be downloaded at: https://www.mdpi.com/article/10.3390/biology15090689/s1, Figure S1. Determination of the optimal threshold for dichotomizing the fucosylation score in colorectal cancer. Table S1: Supplementary Materials S1, Table S2: Supplementary Materials S2, Table S3: Supplementary Materials S3, Table S4: Supplementary Materials S4, Table S5: Supplementary Materials S5, Table S6: Supplementary Materials S6; Table S7: Supplementary Materials S7.
