# Supplementary material for: Fucosylation Dynamics as a Critical Determinant of Cancer Cell Fate in Colorectal Carcinoma: Integrating Hallmark Plasticity, Microenvironmental Remodelling, and Therapeutic Resistance
Source: Biology (Basel). 2026 Apr 28;15(9):689. doi: 10.3390/biology15090689 (PMC13163022; doi:10.3390/biology15090689)
Supplement: Supplementary file 1 [file biology-15-00689-s001.zip › Supplementary Figure S1.pdf]

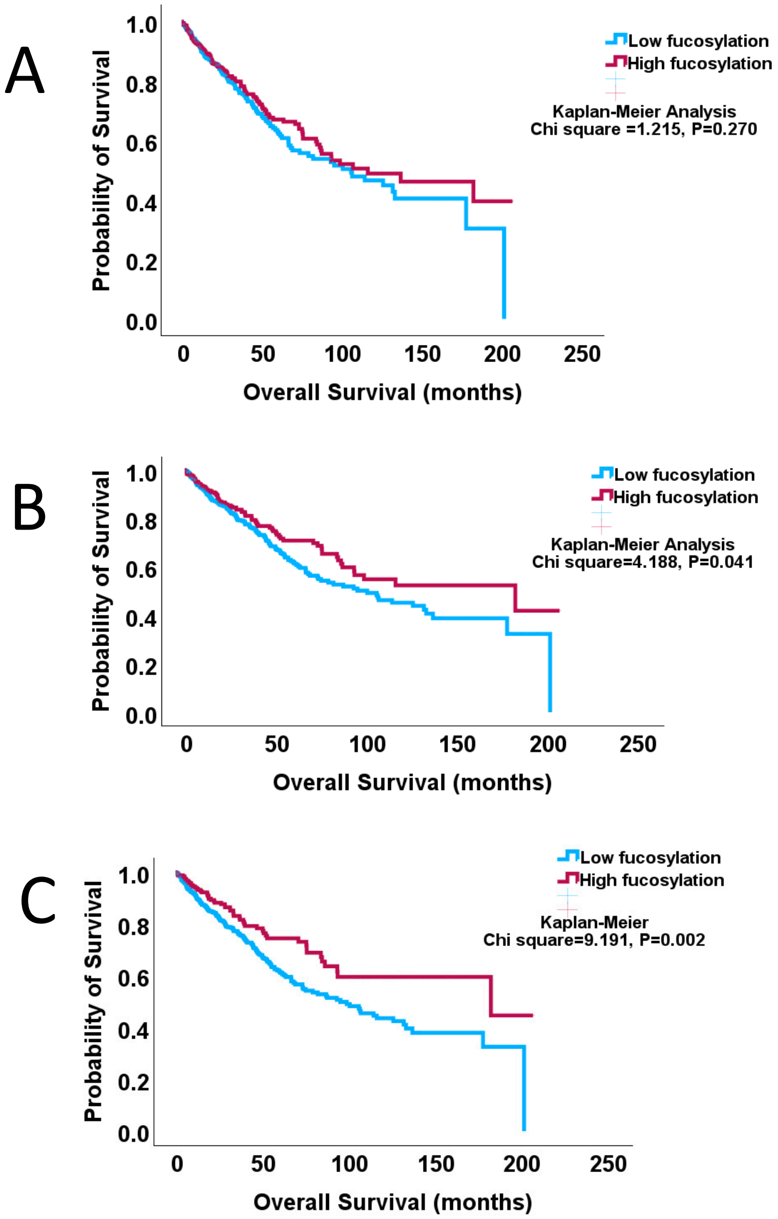

Supplementary Figure S1. Determination of the optimal threshold for dichotomizing the fucosylation score in colorectal cancer. Kaplan–Meier survival analyses were performed using three candidate thresholds for dichotomizing tumour fucosylation scores: the median (50th percentile), 67th percentile, and 75th percentile. For each cutoff, patients were stratified into fucosylation-high and fucosylation-low groups, and differences in overall survival were evaluated using the log-rank (χ^2) test. Among the tested thresholds, the 75th percentile cutoff produced the strongest association with overall survival, as indicated by the highest chi-square statistic and lowest p-value, and was therefore selected as the threshold for defining high- and low-fucosylation subsets in subsequent analyses.
